# 3D Exploration of the Brainstem in 50-Micron Resolution MRI

**DOI:** 10.3389/fnana.2020.00040

**Published:** 2020-09-23

**Authors:** Richard Jarrett Rushmore, Peter Wilson-Braun, George Papadimitriou, Isaac Ng, Yogesh Rathi, Fan Zhang, Lauren Jean O’Donnell, Marek Kubicki, Sylvain Bouix, Edward Yeterian, Jean-Jacques Lemaire, Evan Calabrese, G. Allan Johnson, Ron Kikinis, Nikos Makris

**Affiliations:** ^1^Departments of Psychiatry and Neurology, Center for Morphometric Analysis, A. A. Martinos Center for Biomedical Imaging, Massachusetts General Hospital, Boston, MA, United States; ^2^Psychiatric Neuroimaging Laboratory, Brigham and Women’s Hospital, Boston, MA, United States; ^3^Department of Anatomy and Neurobiology, Boston University School of Medicine, Boston, MA, United States; ^4^Department of Radiology, Brigham and Women’s Hospital, Harvard Medical School, Boston, MA, United States; ^5^Laboratory for Mathematics and Imaging, Brigham and Women’s Hospital, Boston, MA, United States; ^6^Surgical Planning Laboratory, Department of Radiology, Brigham and Women’s Hospital, Boston, MA, United States; ^7^Department of Psychology, Colby College, Waterville, ME, United States; ^8^Service de Neurochirurgie, CHU Clermont-Ferrand, Universite Clermont Auvergne, CNRS, SIGMA Clermont, Clermont-Ferrand, France; ^9^Department of Radiology, Center for In Vivo Microscopy, Duke University Medical Center, Durham, NC, United States; ^10^Computer Science Department, University of Bremen, Institutsleiter, Fraunhofer MEVIS, Bremen, Germany

**Keywords:** ultrahigh-resolution MRI, human brainstem anatomy, neuroimaging, ontology, terminologia anatomica, brainstem atlas

## Abstract

The brainstem, a structure of vital importance in mammals, is currently becoming a principal focus in cognitive, affective, and clinical neuroscience. Midbrain, pontine and medullary structures serve as the conduit for signals between the forebrain and spinal cord, are the epicenter of cranial nerve-circuits and systems, and subserve such integrative functions as consciousness, emotional processing, pain, and motivation. In this study, we parcellated the nuclear masses and the principal fiber pathways that were visible in a high-resolution T2-weighted MRI dataset of 50-micron isotropic voxels of a postmortem human brainstem. Based on this analysis, we generated a detailed map of the human brainstem. To assess the validity of our maps, we compared our observations with histological maps of traditional human brainstem atlases. Given the unique capability of MRI-based morphometric analysis in generating and preserving the morphology of 3D objects from individual 2D sections, we reconstructed the motor, sensory and integrative neural systems of the brainstem and rendered them in 3D representations. We anticipate the utilization of these maps by the neuroimaging community for applications in basic neuroscience as well as in neurology, psychiatry, and neurosurgery, due to their versatile computational nature in 2D and 3D representations in a publicly available capacity.

## Introduction

The human brainstem, a relatively small part of the human brain (roughly the size of a human thumb) yet of utmost biological importance, remains one of the most understudied brain regions in basic and clinical neuroscience. Phylogenetically, the brainstem is the oldest part of the brain. It has three main structural divisions rostrocaudally, commonly referred to as the midbrain (or mesencephalon), pons (Latin for “bridge”) and medulla oblongata (“oblong marrow” as well as “bulbus” in Latin; e.g., Willis, 1681; Carpenter and Sutin, 1983; Nieuwenhuys, 2008; Swanson, 2015)^1^. Located in the posterior cranial fossa between the spinal cord and the diencephalon, the brainstem appears between the 5th and 7th week of embryonic life in humans (Nolte et al., 2016). It is strategically positioned as a conduit for ascending and descending fiber tracts and serves cranial nerve-related as well as higher integrative and neuromodulatory functions (Carpenter and Sutin, 1983; Duvernoy, 1995; Nieuwenhuys, 2008; Blessing and Benarroch, 2012; Nolte et al., 2016). These functions are reflected by the fine morphological, topographical, and qualitative structural architecture of the brainstem, namely the cellular composition of individual nuclei and their fiber connections. From a neural systems perspective, specialized functions of the brainstem are most clearly understood for sensory and motor systems, for which the midbrain, pons, and medulla oblongata contain key interrelated structures. By contrast, the structural and functional anatomy of brainstem systems integrating consciousness, pain, and different types of affect is less clear. Brainstem cytoarchitecture and subsequently its myeloarchitecture have been described to a considerable extent since the early 1900s in classical studies that paved the way for more recent mapping of its nuclear masses and fiber connections (e.g., Olszewski and Baxter, 1982; Swanson, 2015). The atlases resulting from such investigations have enabled us to determine the location and cellular composition of individual nuclei as well as their fiber pathways in the human brainstem. These lines of investigation have been crucial in understanding normative neuroanatomy as well as neuropathology (e.g., Olszewski and Baxter, 1982).

With the advent of MRI, studies of the brainstem have demonstrated the potential of neuroimaging for analyzing this brain component structurally, metabolically and functionally (Toga and Mazziotta, 2002; Aggarwal et al., 2013; Deistung et al., 2013; Beissner, 2015; Sclocco et al., 2018; Tang et al., 2018; Edlow et al., 2019). Currently, *ex-vivo* acquisitions of human brain tissue using high field MRI scanners have allowed for high spatial resolution brainstem datasets (e.g., Calabrese et al., 2015; Edlow et al., 2019). These datasets have enabled the visualization of gray and white matter structures to such a degree that the goal of matching morphologically histological preparations traditionally used in neuroanatomy with those of the MRI images has been met for certain nuclei as a whole, although not yet at the cellular level. Precise MRI-based 2D and 3D computational reconstructions of gray and white matter anatomical structures allow their integration into current multimodal neuroimaging, a unique feature of current morphometric analysis, with a significant advantage over traditional histological methodologies. Consequently, these data can be used in different fields of basic and clinical neuroscience in which knowledge of structure, function, and metabolism of the human brainstem is needed.

In the present study, we parcellated the anatomical gray matter structures and the fiber pathways of conduit and cranial nerve systems that were visible in an ultrahigh-resolution T2-weighted MRI dataset of a postmortem human brainstem. Based on our initial analyses, we combined the gray and white matter parcellation results to generate a detailed map of the human brainstem. To evaluate the anatomical accuracy and precision of our maps, we compared our observations with histological maps of classical human brainstem atlases. Furthermore, by assembling the different component structures, we reconstructed the motor, sensory, and integrative neural systems of the brainstem and rendered them in 3D representations. Given the computational nature of our maps in 2D and 3D spaces, we anticipate their utilization by the neuroimaging community at large for applications in basic and clinical neuroscience.

## Materials and Methods

### Image Acquisition and Protocol Parameters of an Ultrahigh-Resolution *ex vivo* T2-Weighted MRI Dataset

Postmortem imaging was performed in a single human brainstem from an anonymous 65-year old male donor as described by Calabrese et al. (2015), following our institutional rules and guidelines. Briefly, the brain was extracted with a post-mortem interval of 24 h and the brainstem and thalamus were dissected. The vasculature was flushed with normal heparinized saline (100 UI/ml heparin) and the brain subsequently immersed in 10% neutral buffered formalin for 2 weeks. A week before scanning, the sample was placed in a 0.1 M phosphate buffer containing 1% gadoteridol. Before imaging, the brain was immersed in liquid fluorocarbon (Galden PFPE, Solvay Plastics, Brussels, Belgium). RF transmission and reception were achieved with a 65 mm inner-diameter quadrature RF coil. Anatomical images were acquired using a 3D gradient echo pulse sequence with repetition time (TR) = 50 ms, echo time (TE) = 10 ms, flip angle (a) = 60 degrees, and bandwidth (BW) = 78 Hz/pixel. The field of view (FOV) was 80 × 55 × 45 mm, and the acquisition matrix was 1,600 × 1,100 × 900 resulting in a 50-micrometer isotropic voxel size. The total acquisition time was 14 h (Calabrese et al., 2015).

### Anatomical Image Analysis: Segmentation and Labeling of Regions of Interest

Identification and delineation of anatomical regions of interest (ROI) were carried out on the T2-weighted MRI dataset as follows. Every 5th axial slice was manually segmented, using the 3D Slicer software analysis platform to perform segmentation and labeling of visualized brainstem anatomical structures (Fedorov et al., 2012). All the tools used are publicly available and can be downloaded from the official 3D Slicer website^1^. The segmentation of structures was done as follows. The brainstem was segmented as a whole and then subdivided into three component parts, namely midbrain or mesencephalon, pons, and medulla. Following traditional anatomical conventions, the limiting axial plane between the midbrain and the pons was set at the uppermost extent of the basis pontis, whereas the limiting axial plane between the pons and the medulla was set at the lowermost extent of the basis pontis (DaSilva et al., 2002). This yielded a gross morphological definition and topographical delimitation of the brainstem in the context of traditional neuroanatomical descriptions. Subsequently, segmentation of gray matter and white matter structures was carried out individually for each nucleus and fiber tract. This was performed using the Segment Editor module of 3D Slicer, which allows segmentation operations. To ensure anatomical accuracy in the identification of gray and white matter structures we were also guided by anatomical textbooks and atlases of the brainstem. These included Olszewski-Baxter (Olszewski and Baxter, 1954, 1982; Büttner-Ennever and Horn, 2014) Paxinos and Huang (Paxinos and Huang, 1995), Haines (Haines, 1991), Carpenter and Sutin (Carpenter and Sutin, 1983), Mai and Paxinos (Mai and Paxinos, 2012), and Nolte (Nolte et al., 2016; Vanderah, 2018). Furthermore, we assessed our results by matching the extent of each structure of our analysis with those of Paxinos and Huang (1995). To address neural systems analysis in the brainstem, we assembled individual structures into the motor, sensory and integrative (or neuromodulatory) neural systems, a topic of great relevance in current basic and clinical neuroscience (Dahlström and Fuxe, 1964; Mesulam, 2000; Solms and Turnbull, 2002; Panksepp, 2004; Damasio, 2010; Blessing and Benarroch, 2012).

## Results

**Figure 1:**
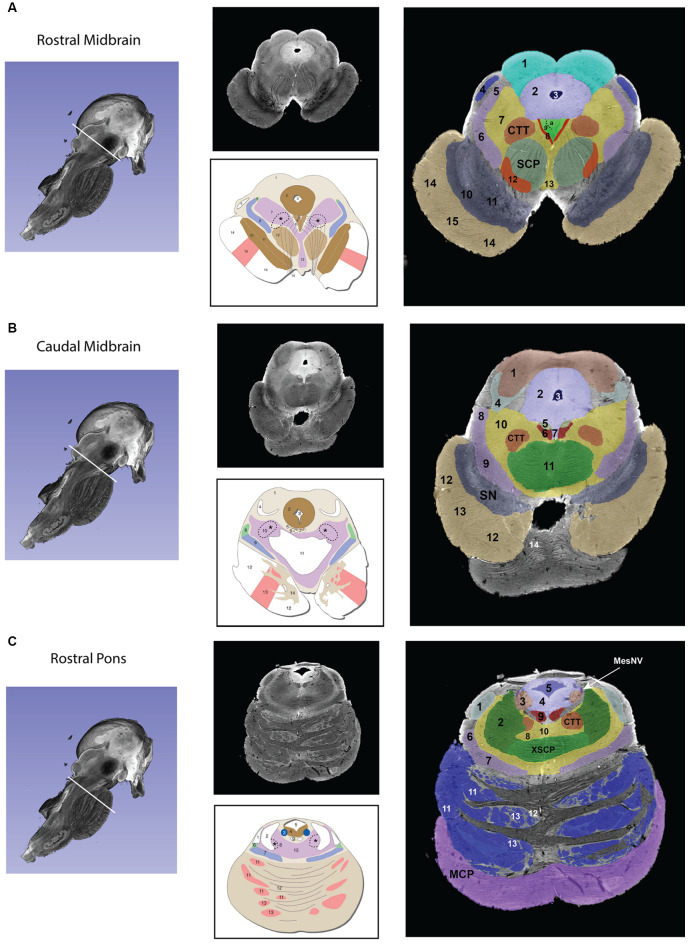

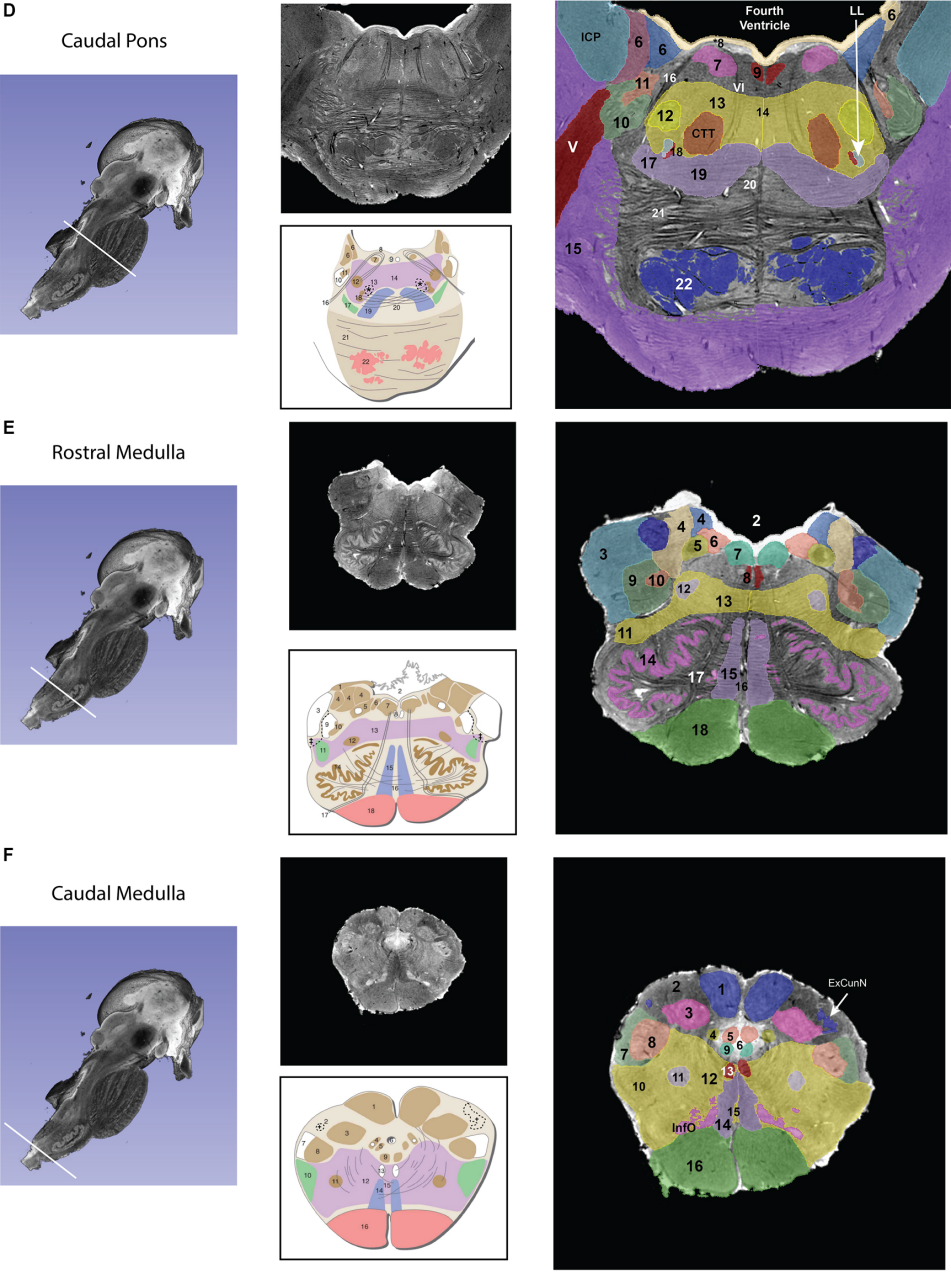
Transverse segmented sections through the midbrain **(A,B)**, pons **(C,D)**, and medulla **(E,F)** of the brainstem. For each subfigure, a hemisected longitudinal view of the brainstem is shown on the left with a white line representing the plane of the section that corresponds to the images in the middle panel. The upper section of the middle panel is an image from the MRI dataset, and the lower picture is a schematic image from Essentials of the Human Brain (Vanderah, 2018) of a comparable brainstem section. The numbers in the schematic figures are recapitulated in the large segmented image on the right to show the correspondence of structures between the MRI dataset and the schematic image. Note that: (1) in some instances, some numbered structures present in the schematic figures do not appear in the segmented MRI image because they could not be reliably identified in the MRI image; and (2) some structures appear in the MRI image but not in the schematic images—these structures are identified by the alphabetical abbreviation and reflect slight differences in the angle of cut or structures that were clear in the MRI but not demarcated in the schematic images. The red nucleus, in particular, appears minimally in the MRI image due to the angle of the section, which was taken at the caudal-most tip of the red nucleus. More specifically, this section represents the level where fibers of the superior cerebellar peduncle enter in the red nucleus. Furthermore, the central tegmental tract, which does not appear in the schematic figure has been identified in the MRI image. Moreover, fibers of the oculomotor nerve traversing the superior cerebellar peduncle are visible in both the MRI image and the schematic figure. Figures from Vanderah (2018) used with permission. Abbreviations: **(A)** 1. Superior Colliculus, 2. Periaqueductal gray, 3. Cerebral Aqueduct, 4. Brachium of the inferior colliculus, 5. Anterolateral system, 6. Medial lemniscus, 7. The reticular formation, 8. Medial longitudinal fasciculus, 9. Oculomotor nuclear complex (a. Nucleus of Edinger-Westphal), 10. Substantia Nigra pars reticulata, 11. Substantia nigra pars compacta, 12. Red nucleus, 13. Ventral tegmental area, 14. Corticopontine fibers of the Cerebral peduncle, 15. Corticospinal and corticobulbar fibers of the Cerebral peduncle. CTT—Central tegmental tract, SCP—Superior cerebellar peduncle. The CTT was added in the schematic image from Essentials of the Human Brain (Vanderah, 2018) in a dashed line indicated by an asterisk. **(B)** 1. Inferior colliculus, 2. Periaqueductal gray, 3. Cerebral aqueduct, 4. Lateral lemniscus, 5. Trochlear nucleus, 6. Medial longitudinal fasciculus, 7. Raphe nuclei, 8. Anterolateral system, 9. Medial lemniscus, 10. The reticular formation, 11. Decussation of the superior cerebellar peduncle 12. Corticopontine fibers of the Cerebral peduncle, 13. Corticospinal and corticobulbar fibers of the Cerebral peduncle, 14. Pontine nuclei. CTT—Central tegmental tract, SN—Substantia nigra. The CTT was added in the schematic image from Essentials of the Human Brain (Vanderah, 2018) in a dashed line indicated by an asterisk. **(C)** 1. Lateral lemniscus, 2. Superior cerebellar peduncle, 3. Locus coeruleus, 4. Periaqueductal gray, 5. Fourth ventricle, 6. Anterolateral system, 7. Medial lemniscus, 8. The reticular formation, 9. Medial longitudinal fasciculus, 10. Raphe nuclei, 11. Corticopontine fibers of the Cerebral peduncle, 12. Pontine nuclei, 13. Corticospinal and corticobulbar fibers. CTT—Central tegmental tract, MCP—Middle cerebellar peduncle, MesNV—Mesencephalic tract and nucleus of trigeminal, XSCP—Decussation of the superior cerebellar peduncle. The CTT was added in the schematic image from Essentials of the Human Brain (Vanderah, 2018) in a dashed line indicated by an asterisk. **(D)** 6. Vestibular complex of nuclei, 7. Abducens nucleus, *8. The location of the internal genu of the facial nerve is indicated here, although it should be noted that in this MRI image the plane of the section includes both fibers of the facial nerve laterally (16 in this figure) and fibers of the abducens nerve medially (VI), 9. Medial longitudinal fasciculus, 10. Spinal tract of the trigeminal, 11. Spinal nucleus of the trigeminal, 12. Facial motor nucleus, 13. Reticular formation, 14. Raphe nuclei, 15. Middle cerebellar peduncle, 16. Facial nerve fibers, 17. Anterolateral system, 18. Superior olivary nucleus, 19. Medial lemniscus, 20. Trapezoid body, 21. Pontine nuclei, 22. – Corticospinal, corticobulbar, and corticopontine fibers. CTT—Central tegmental nucleus, ICP—Inferior cerebellar peduncle, LL—Lateral lemniscus, MCP—Middle cerebellar peduncle, V—Trigeminal nerve fibers, VI—Abducens nerve fibers. The CTT was added in the schematic image from Essentials of the Human Brain (Vanderah, 2018) in a dashed line indicated by an asterisk. **(E)** 2. Fourth Ventricle, 3. Inferior cerebellar peduncle, 4. Vestibular nuclei, 5. Solitary tract and nucleus of the solitary tract, 6. Dorsal motor nucleus of the vagus, 7. Hypoglossal nucleus, 8. Medial longitudinal fasciculus, 9. Spinal trigeminal tract, 10. Spinal trigeminal nucleus, 11. Anterolateral system, 12. Nucleus ambiguous, 13. Reticular formation, 14. Inferior olivary nucleus, 15. Medial lemniscus, 16. Raphe nuclei, 17. Hypoglossal nerve fibers, 18. Pyramid. Please note that in the MRI image the cochlear nuclei are not present due to the plane of section. The border of the inferior cerebellar peduncle was extended in the schematic image from Essentials of the Human Brain (Vanderah, 2018) in a dashed line indicated by a double-cross. **(F)** 1. Nucleus gracilis, 2. Fasciculus cuneatus, 3. Nucleus cuneatus, 4. Solitary tract and nucleus of the solitary tract, 5. Dorsal motor nucleus of the vagus, 7. Spinal trigeminal tract, 8. Spinal trigeminal nucleus, 9. Hypoglossal nucleus, 10. Anterolateral system, 11. Nucleus ambiguous, 12. Reticular formation, 13. Medial longitudinal fasciculus, 14. Medial lemniscus, 15. Raphe nuclei, 16. Pyramid, InfO—Inferior olivary nucleus. The external cuneate nucleus was added in the schematic image from Essentials of the Human Brain (Vanderah, 2018) in a dashed line indicated by a single cross.

**Figure 2:**
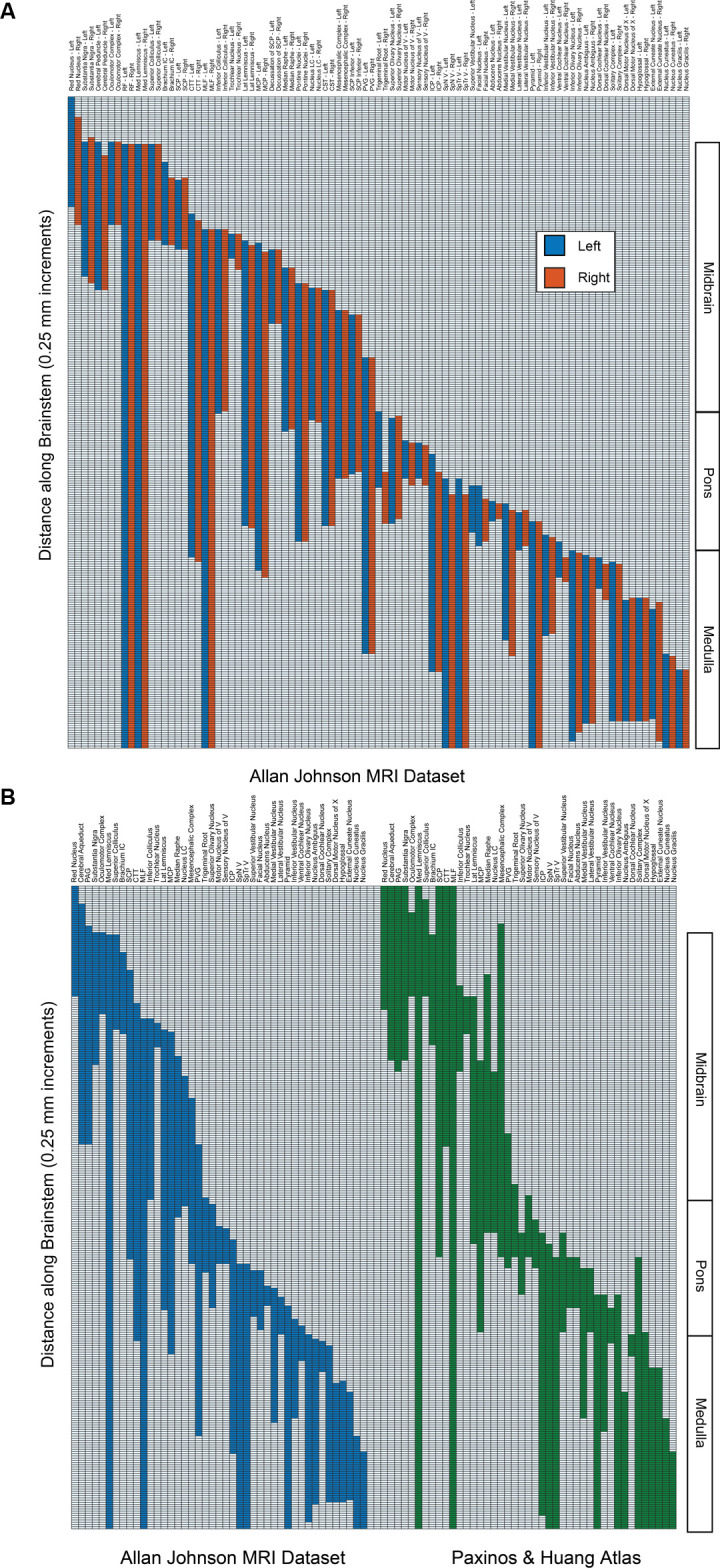
**(A)** The superior-inferior extent of brainstem structures on the left (blue) and right (red) from the present dataset. Regions are indicated on the abscissa, and the presence of the regions from superior (upper) to inferior (lower) is indicated in 250-micrometer increments on the ordinate. The right-hand side indicates the divisions of the brainstem into midbrain, pons, and medulla. Note that the unpaired cerebral aqueduct and periaqueductal gray are not included. **(B)** Comparison of the superior-inferior extent of brainstem structures from the present dataset (Allan Johnson, blue) with the Paxinos and Huang (1995) atlas (green). All conventions are as in 2A. Note that some regions that were identified in the present dataset (i.e., reticular formation, pontine nuclei, corticospinal tract, cerebral peduncle) were not identified in the *Paxinos and Huang* atlas *per se* and were removed for purposes of clarity.

**Figure 3:**
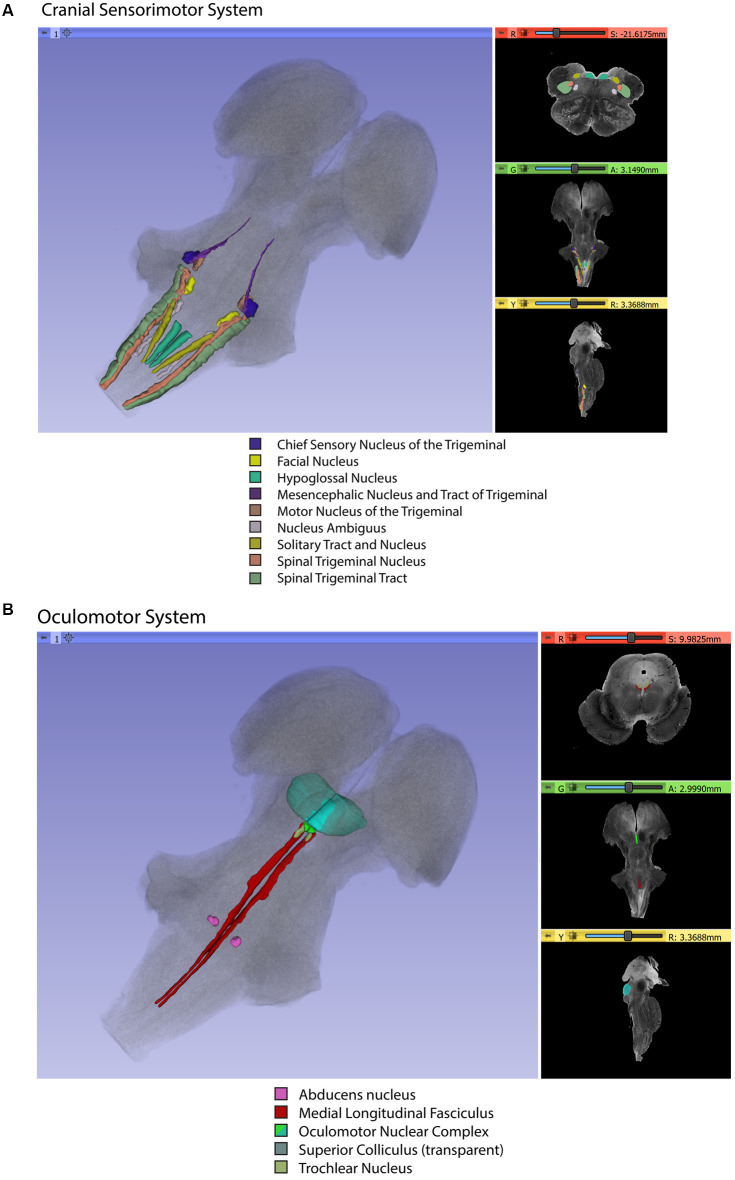

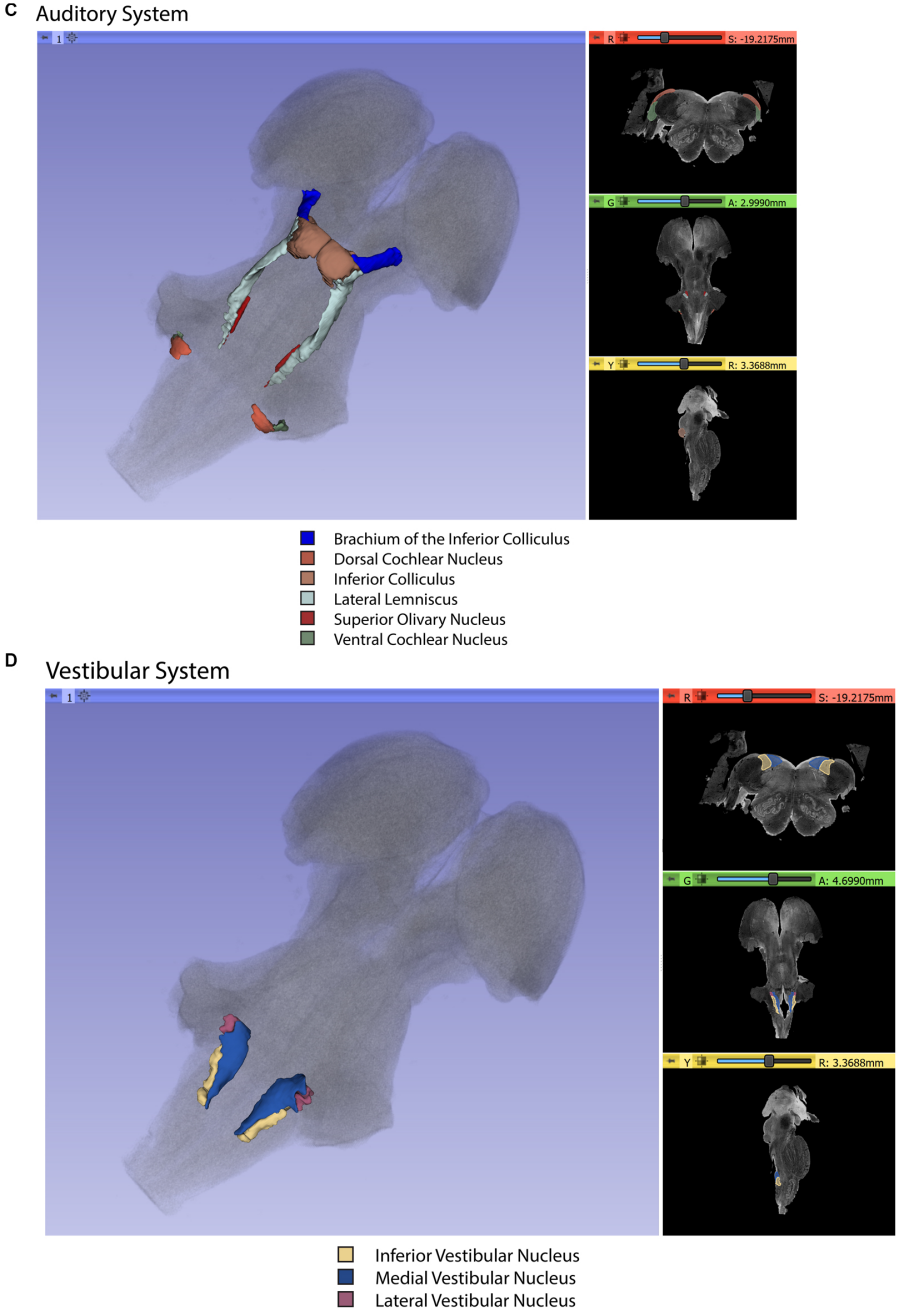

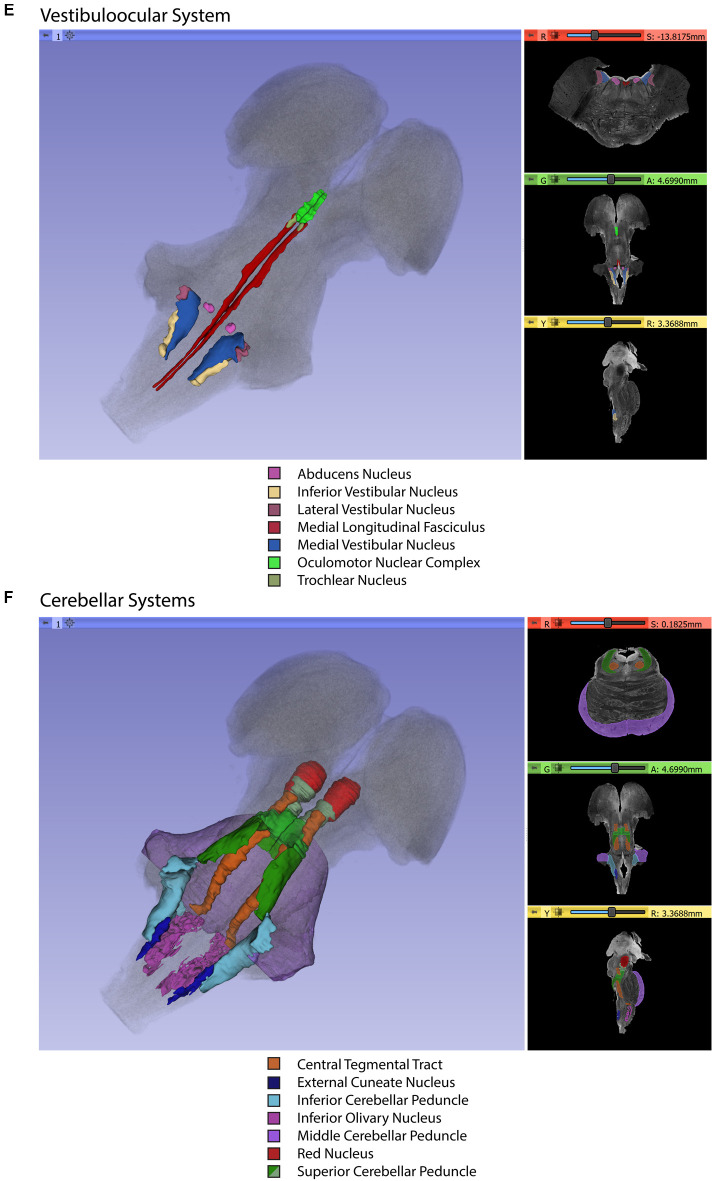

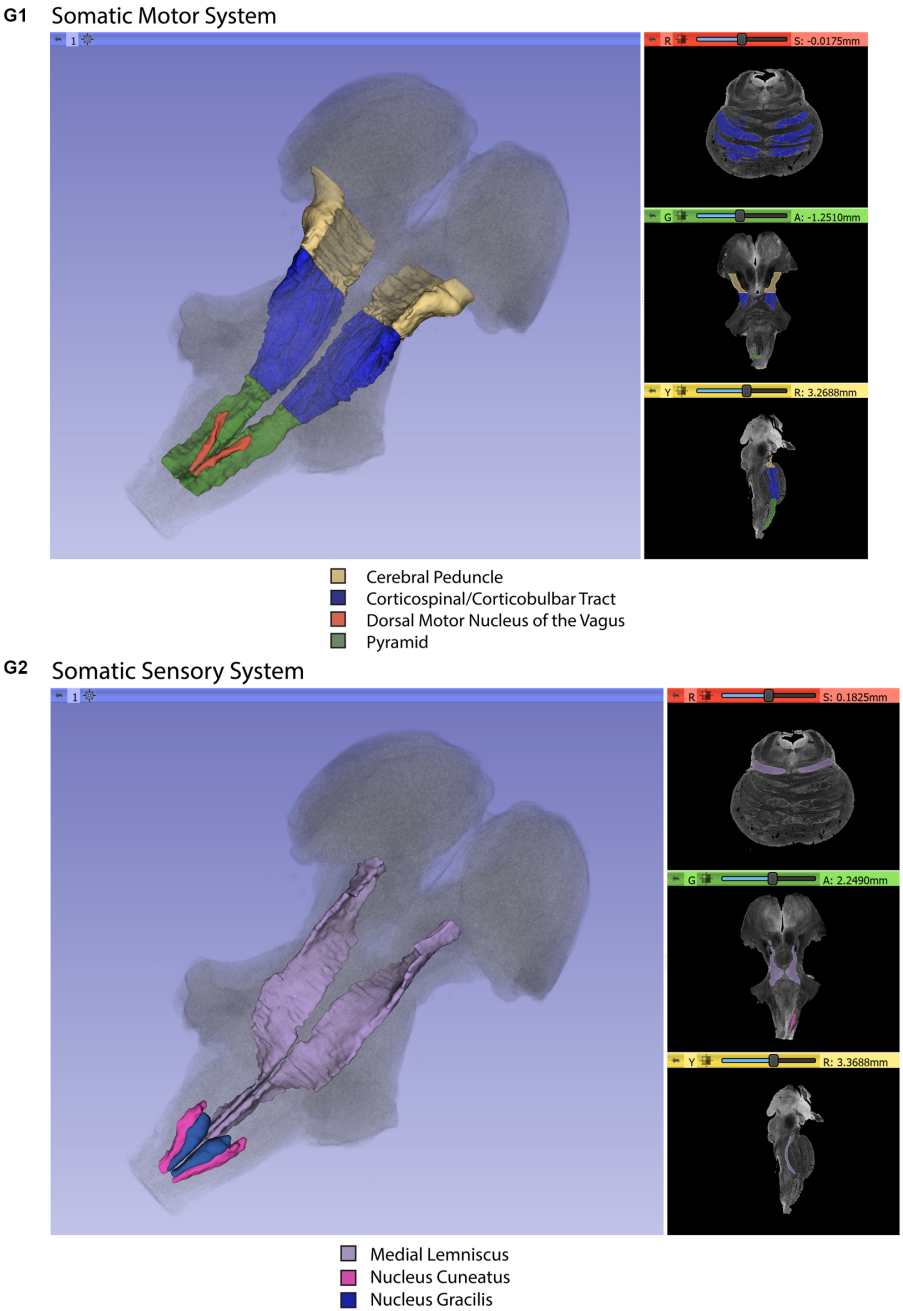

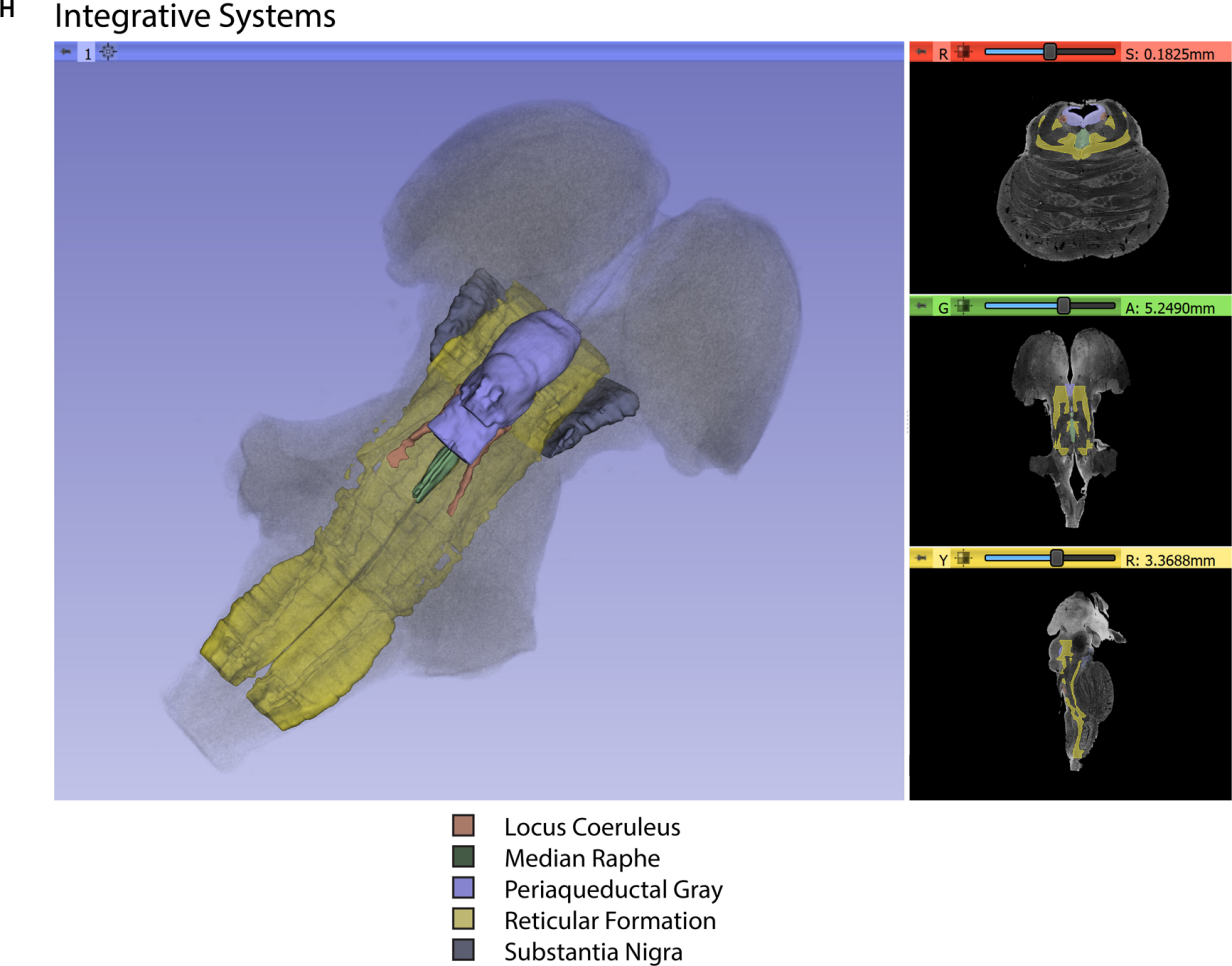
Three-dimensional reconstructions of selected brainstem systems. For each subfigure, the three-dimensional rendering is overlaid on a volume rendering of the brainstem, and selected slices in the axial (red), coronal (yellow), and sagittal (green) planes are illustrated on the right. To generate the three-dimensional structures, a 0.5 mm median smoothing kernel was applied. **(A)** Cranial sensorimotor system, **(B)** Oculomotor system, **(C)** Auditory system, **(D)** Vestibular system, **(E)** Vestibuloocular system, **(F)** Cerebellar systems, **(G)** Motor **(G1)** and Sensory **(G2)** systems of the body, **(H)** Integrative systems. Note that the most easily resolvable structures on the MRI images were segmented and reconstructed and that these images do not encapsulate all component structures of each system.

Gross segmentation of the brainstem reflecting its three principal parts, namely midbrain, pons, and medulla, was readily accomplished. At an individual structure level of analysis, we were able to identify 47 gray and white matter structures within the entire brainstem and the cerebral aqueduct. These consisted of 25 gray matter structures, 22 fiber tracts as well as the cerebral aqueduct. Of the 47 structures identified, we were able to delineate morphometrically all 25 gray matter structures, 16 white matter structures (including the trigeminal root), and the cerebral aqueduct as listed in detail in Table 1 and illustrated in Figure 1 in six representative axial sections. For anatomical reference and clarity, we displayed our results side-by-side with corresponding plates of the Nolte textbook (Nolte et al., 2016; Vanderah, 2018). The latter was done for didactic purposes to aid the student of brainstem anatomy who is not sufficiently familiar with this complex structure. The results observed in our analysis matched the topography reported by classical atlases, namely those of Haines (1991), Paxinos and Huang (1995), and Tona et al. (2017). The size of our brainstem dataset (i.e., 54.2 mm in length) was comparable to the sample used by Tona et al. (2017; i.e., 54.02 mm) by 99.7%, and with the brainstem analyzed by Paxinos and Huang (1995; i.e., 59 mm) by 91.6%. The topographical relationships among the various gray and white matter structures and the ventricular complex also met expectations based on traditional neuroanatomy. More specifically, a comparison between left and right structures in our analysis showed an overall 99% correspondence and a spatial overlap of 97%, as shown in Figure 2A. Moreover, the overall segmentation of individual ROIs showed a correspondence of 88% and an overall spatial overlap of our segmentations of 87% as compared to Paxinos and Huang (1995). The latter comparison is represented numerically in Table 2 and graphically in Figure 2B. The histological sectioning and processing procedures used to generate the atlases (Olszewski and Baxter, 1982; Paxinos and Huang, 1995) are likely the main source of the differences in the overlap between the atlases and the present material. At another level of analysis, the neural systems associated with brainstem structures were delineated and reconstructed in three-dimensional space as shown in Figure 3. These were the motor and sensory systems of the face (Figure 3A), the oculomotor system (Figure 3B), the auditory system (Figure 3C), the vestibular system (Figure 3D), the vestibuloocular system (Figure 3E), the cerebellar systems (Figure 3F), the motor and sensory body systems (Figures 3G1,G2) and the integrative systems (Figure 3H). Finally, we produced a full segmentation as a set of 291 axial atlas plates in the Supplementary Material.

**Table 1:** List of gray and white matter structures to relate brainstem structures as defined by Nolte et al. (2016) and Vanderah (2018) with the present study and with the terminologia neuroanatomica (ten Donkelaar et al., 2017, 2018; FIPAT.library.dal.ca).

	Gray matter structures		
Nolte	Present study	Terminologia neuroanatomica (TNA)	TA98 ID
Abducens nucleus	Abducens nucleus	Nucleus nervi abducentis	A14.1.05.411
Cochlear nuclei	Dorsal cochlear nucleus Ventral cochlear nucleus	Nucleus cochlearis posterior Nucleus cochlearis anterior	A14.1.04.248 A14.1.04.249
Dorsal motor nucleus of the vagus	Dorsal motor nucleus of X	Nucleus posterior nervi vagi	A14.1.04.229
Edinger-Westphal nucleus	Oculomotor complex	Nucleus accessorii nervi oculomotorii	A14.1.06.303
External cuneate nucleus	External cuneate nucleus	Nucleus cuneatus accessories	A14.1.04.209
Facial motor nucleus	Facial nucleus	Nucleus nervi facialis	A14.1.05.412
Hypoglossal nucleus	Hypoglossal	Nucleus nervi hypoglossi	A14.1.04.227
Inferior colliculus	Inferior colliculus—Anatomical Inferior colliculus—CN	Colliculus inferior	A14.1.06.014
Inferior olivary nucleus	Inferior olivary nucleus	Nucleus olivaris principalis	A14.1.04.220
Locus coeruleus	Nucleus locus coeruleus	Nucleus caeruleus	A14.1.05.436
Nucleus ambiguus	Nucleus ambiguus	Nucleus ambiguus	A14.1.04.253
Nucleus cuneatus	External cuneate nucleus Nucleus cuneatus	Nucleus cuneatus	A14.1.04.206
Nucleus gracilis	Nucleus gracilis	Nucleus gracilis	A14.1.04.202
Nucleus of the solitary tract	Solitary complex	Nuclei tractus solitarii	A14.1.04.230
Oculomotor nucleus	Oculomotor complex	Nucleus nervi oculomotorii	A14.1.06.302
Periaqueductal gray	PAG	Substantia grisea periaquaeductalis	A14.1.06.321
Periventricular gray	PVG	
Pontine nuclei and pontocerebellar fibers	Pontine nuclei	Nuclei pontis	A14.1.05.202
Raphe nuclei	Median raphe Dorsal raphe	Nucleus raphes medianus Nucleus raphes dorsalis	A14.1.05.402 A14.1.05.601 A14.1.06.401
Red nucleus	Red nucleus	Nucleus ruber	A14.1.06.323
Spinal trigeminal nucleus	SpN V	Nucleus spinalis nervi trigemini	A14.1.05.404 A14.1.04.211
Motor nucleus of the trigeminal	Motor nucleus of V	Nucleus motorius nervi trigemini	A14.1.05.410
Sensory nucleus of the trigeminal	Sensory nucleus of V	Nucleus principalis nervi trigemini	A14.1.05.406
Mesencephalic nucleus of the trigeminal	Mesencephalic complex	nucleus mesencephalicus nervi trigemini	A14.1.05.409
Substantia nigra	Substantia nigra	Substantia nigra	A14.1.06.111
Superior colliculus	Superior colliculus	Colliculus superior	A14.1.06.015
Superior olivary nucleus	Superior olivary nucleus	Complexus olivaris superior	A14.1.05.415
Trochlear nucleus	Trochlear nucleus	Nucleus nervi trochlearis	A14.1.06.319
Vestibular nuclei	Inferior vestibular nucleus Lateral vestibular nucleus Medial vestibular nucleus Superior vestibular nucleus	Nucleus vestibularis inferior Nucleus vestibularis lateralis Nucleus vestibularis medialis Nucleus vestibularis superior	A14.1.04.243 A14.1.05.427 A14.1.05.426 A14.1.05.429
**White matter structures**
Brachium of the inferior colliculus	Brachium IC	Brachium colliculi inferioris	A14.1.06.012
	CTT	Tractus tegmentalis centralis	A14.1.05.325
Cerebral peduncle	Cerebral peduncle	Pedunculus cerebri	A14.1.06.004
Decussation of the superior cerebellar peduncles	Decussation of SCP	Decussatio pedunculorum cerebellarium superiorum	A14.1.06.217
Inferior cerebellar peduncle	ICP	Pedunculus cerebellaris inferior	A14.1.07.413/ A14.1.04.013
Lateral lemniscus	Lat lemniscus	Lemniscus lateralis	A14.1.05.317 A14.1.06.204 A14.1.08.670
Medial lemniscus	Med lemniscus	Lemniscus medialis	A14.1.04.111 A14.1.06.207 A14.1.08.672
Medial longitudinal fasciculus	MLF	Fasciculus longitudinalis medialis	A14.1.05.304 A14.1.04.113 A14.1.06.209
Middle cerebellar peduncle	MCP	Pedunculus cerebellaris medius	A14.1.07.416 A14.1.05.003
Pyramid	Pyramid	Pyramis medullae oblongatae	A14.1.04.003
Reticular formation	RF	Formatio reticularis	A14.1.00.021 A14.1.05.403 A14.1.06.327
Solitary tract	Solitary complex	Tractus solitarius/Nucleus tractus solitarii	A14.1.04.120/ A14.1.04.230
Spinal trigeminal tract	SpTr V	Tractus spinalis nervi trigemini	A14.1.05.309 A14.1.04.115
Superior cerebellar peduncle	SCP SCP—Inferior	Pedunculus cerebellaris superior	A14.1.07.417 A14.1.05.006 A14.1.06.216 A14.1.06.009 A14.1.08.678
**Other structures**
Cerebral aqueduct	Cerebral aqueduct	Aquaeductus mesencephali	A14.1.06.501

*The last column contains identification codes for the structures as found in the terminologia anatomica viewer (https://taviewer.openanatomy.org). The trigeminal root is not included here because it does not correspond explicitly to the TNA nomenclature*.

**Table 2:** Ratios of the superior-inferior extent of brainstem structures from the present dataset (Allan Johnson MRI dataset) with the Paxinos and Huang (1995) atlas.

Structure	Ratio	Structure	Ratio
Red Nucleus	0.89	Sensory Nucleus of V	0.77
Cerebral Aqueduct	1.40	ICP	0.76
PAG	1.18	SpN V	0.93
Substantia Nigra	0.76	SpTr V	0.98
Oculomotor Complex	1.16	Superior Vestibular Nucleus	0.47
Medial Lemniscus	0.92	Facial Nucleus	1.17
Superior Colliculus	0.98	Abducens Nucleus	0.39
Brachium IC	0.74	Medial Vestibular Nucleus	0.90
SCP	0.78	Lateral Vestibular Nucleus	0.62
CTT	1.31	Pyramid	0.95
MLF	0.79	Inferior Vestibular Nucleus	0.73
Inferior Colliculus	0.87	Ventral Cochlear Nucleus	1.00
Trochlear Nucleus	0.65	Inferior Olivary Nucleus	0.80
Lateral Lemniscus	1.12	Nucleus Ambiguus	1.21
MCP	1.19	Dorsal Cochlear Nucleus	1.40
Nucleus LC	0.81	Solitary Complex	0.58
Mesencephalic Complex	0.44	Dorsal Motor Nucleus of X	0.60
Median Raphe	0.62	Hypoglossal	0.74
PVG	2.16	External Cuneate Nucleus	0.67
Trigeminal Root	1.03	Nucleus Cuneatus	0.84
Superior Olivary Nucleus	1.68	Nucleus Gracilis	0.90
Motor Nucleus of V	0.61		

*Note that some regions that were identified in the present dataset (i.e., reticular formation, pontine nuclei, corticospinal tract, cerebral peduncle) were not identified in the Paxinos and Huang atlas *per se* and were removed for purposes of clarity. Abbreviations: CTT, central tegmental tract; IC, inferior colliculus; ICP, inferior cerebellar peduncle; LC, locus coeruleus; MCP, middle cerebral peduncle; MLF, medial longitudinal fasciculus; PAG, periaqueductal gray; PVG, periventricular gray; SCP, superior cerebellar peduncle; SpNV, the spinal nucleus of the trigeminal; SPTrV, spinal tract of the trigeminal; X, vagus*.

## Discussion

In this study, we localized and delineated the principal structures of the human brainstem using an MRI *ex vivo* ultrahigh-resolution dataset with properties largely comparable to histological representation. More specifically, we volumetrically identified 47 gray (viz., 25) and white matter (viz., 22) structures in the brainstem based on an *ex vivo* human T2-weighted MRI dataset of 50-micron spatial resolution. Furthermore, we were able to delineate morphometrically all 25 gray matter structures and 16 of the 22 white matter structures. To our knowledge, this has not been previously achieved in MRI-based morphometric analysis. Moreover, we assembled individual structures into neural systems specifically serving cranial nerve, conduit, and integrative functions.

The brainstem is becoming a major focus in clinical and basic neuroscience due to our current ability to image it volumetrically at resolutions previously inaccessible to MRI. It is a structure that appears early in evolution and has been studied structurally across several species (Ariëns-Kappers et al., 1960; Angevine, 1961; Emmers and Akert, 1963; Berman, 1968; Madigan and Carpenter, 1971; Paxinos and Huang, 1995; Franklin and Paxinos, 1997; Nieuwenhuys et al., 1998; Paxinos, 1999; Paxinos et al., 1999, 2000; Naidich et al., 2009; Baizer, 2014). Although, relatively small in size, this brain structure is associated with vital biological integrative functions such as consciousness, motivation, pain, and reward as well as cranial nerve biology and conduit somatosensory and motor functions. Thus, the brainstem is critical for a complete understanding of brain structure-function relationships. We expect our anatomical analysis to be of use to neuroanatomists as well as clinical and basic neuroscientists.

Early students of neuroanatomy who macrodissected the human brain provided gross, approximate descriptions of the brainstem as a whole and its component parts, namely the midbrain, pons and medulla (e.g., Vesalius, 1543; Varoli, 1573; Diemerbroeck, 1689; Haller, 1747; von Baer, 1837). Microscopic studies of the human brainstem have provided comprehensive descriptions of this structure since the early 1900s. A historical review of these aspects of the human brainstem has been provided by Olszewski and Baxter (1982) and can be highlighted briefly as follows. Jacobsohn’s drawings in particular, given their anatomical accuracy, have served as a classical guideline for anatomical work regarding the brainstem nuclei (Jacobsohn, 1909). The classical work of Ziehen (1903) has provided extensive descriptions of cyto- and myelo-architecture of fiber pathways in the brainstem. Subsequent cytoarchitectonic studies have provided additional detail and more refined visualization with the use of photomicrographs (Gagel and Bodechtel, 1930; Stern, 1935; Crosby and Woodburne, 1943; Riley, 1943). More recently, the atlases of Paxinos and Huang (1995) and Tona et al. (2017) stand out for their in-depth descriptions of brainstem nuclei cytoarchitecture and topography. It should be noted that the Paxinos and Huang (1995) atlas provides unprecedented detail and coverage of not only nuclei but also fiber tracts in the human brainstem—almost twice the number of fiber pathways compared to other atlases. Given that in the present study we needed to simplify and adapt the brainstem nuclear and fiber tract analysis to the neuroimaging data under analysis, we used primarily the Tona et al. (2017) atlas as well as the atlas of Haines (1991), while the Paxinos and Huang (1995) atlas served as the final comparison reference and testbed for our analyses. The results of the analyses were also consistent with Naidich et al. (2009). Finally, for didactic purposes we have displayed the human brainstem gray and white matter structures in six classical axial planes, namely, through the rostral and caudal midbrain, rostral and caudal pons, rostral and caudal medulla, as generally accepted in neuroanatomy following, for example, the Nolte textbook (Nolte and Angevine, 1995; Nolte, 1999; Nolte et al., 2016; Vanderah, 2018).

With the advent of MRI, there have been several studies addressing the *in vivo* and non-invasive visualization of the gray and white matter of the human brainstem (Salamon et al., 2005; Kamali et al., 2009; Yang et al., 2011; Linnman et al., 2012; Bianciardi et al., 2015, 2018; Meola et al., 2016; Sclocco et al., 2018). MRI-based morphometry has examined the morphological and volumetric characterization of the brainstem since the early 1990s (Filipek et al., 1994). Although the gross nature of these investigations precluded their addressing the fine architecture of the brainstem, these early morphological studies indicated the great potential of neuroimaging to visualize this structure *in vivo* and non-invasively. As neuroimaging technology and methods of analysis evolved, brainstem anatomical analysis advanced using structural T1- and T2-weighted MRI as well as diffusion MRI (dMRI) and dMRI tractography. Pioneering morphometric studies with structural imaging measured the major components of the brainstem, namely the midbrain, pons, and medulla, and were used to localize functional activation of specific cranial nerve nuclei in combination with task-specific fMRI acquisitions (e.g., DaSilva et al., 2002). Furthermore, specific nuclear masses have been identified and labeled using T1- and T2-weighted MRI (Linnman et al., 2012; Bianciardi et al., 2015, 2016, 2018; Tona et al., 2017). Moreover, using dMRI tractography, several fiber tract connections have been identified and delineated in the brainstem. Although most studies have addressed principally major motor and sensory connections such as the corticospinal tract (e.g., Salamon et al., 2005; Meola et al., 2016), the cerebellar peduncles (Meola et al., 2016), the corticopontocerebellar pathways (Habas and Cabanis, 2007), and the medial and lateral lemniscus (Kamali et al., 2009; Meola et al., 2016), there are also investigations focusing on finer connections such as the rubrospinal tract (Yang et al., 2011; Meola et al., 2016), spinothalamic tract, medial longitudinal fasciculus, dorsal longitudinal fasciculus (Meola et al., 2016) and central tegmental tract (Kamali et al., 2009; Meola et al., 2016).

In the context of an increasing body of information in the anatomical brainstem imaging field, to our knowledge the present study takes a step forward in identifying and delineating the greatest number of nuclear structures to date. This was achieved principally because of the high spatial resolution, and the very high signal quality of the dataset we analyzed. Importantly, the anatomical analysis was done by expert neuroanatomists using 3D Slicer segmentation and visualization tools. Finally, comparisons of the delineated nuclear structures were done with guidance from classical brainstem textbooks and atlases portraying the precise cytoarchitecture and topography of the human brainstem nuclei. The present analysis represents an advancement concerning traditional histological atlases, given the volumetric nature of our data and our ability to use MRI at a 50-micron resolution. Furthermore, histological slices often have physical artifacts and geometric distortions (e.g., small tears due to tissue folding) and are difficult to reconstruct as a single 3D object. The inherent 3D nature of MRI reduces tissue distortions, leading to more accurate representations in MR-based atlases. Moreover, *in vivo* histology is not possible; thus, relating histological atlases to *in vivo* imaging can be problematic. In contrast, MRI is the primary modality for structural brain imaging, and MR-based atlases appear to be more appropriate and easier to register, and thus to inform more accurately *in vivo* MR-based studies or interventions.

By delineating the structural parts of the brainstem, we have provided a viable means of visualizing in 3D the different cranial nerve systems, conduit systems, and integrative systems. This is a useful method for illustrating and understanding brainstem anatomy for basic neuroscientists as well as neurologists, psychiatrists, and neurosurgeons. As a result of this endeavor, 3D visualizations in the publicly available platform of 3D Slicer allow the student of the brainstem to use the atlas provided herein readily and relatively simply as a learning and teaching tool for this highly complex domain of neuroanatomy.

### Functional Considerations

Brainstem functions can be generally categorized in a didactic, simplistic manner as conduit, cranial nerve, and integrative. The topographic arrangement of the brainstem with the spinal cord, cerebellum, and cerebrum makes it a natural route of passage for the numerous fiber tracts interconnecting these structures, thus justifying its role as a *conduit of fibers of passage*. In this study, we labeled 22 and delineated 16 of these fiber pathways, as shown in Table 1 and Figures 1, 3. It should be noted, however, that fibers of passage may also course through the nuclei of the midbrain, pons, and medulla. Although three of the *cranial nerves*, namely olfactory (cranial nerve I), optic (cranial nerve II), and accessory (cranial nerve XI), do not project primarily or directly to the brainstem, the other nine are anatomically associated with the brainstem. Herein, we were able to label the majority of cranial nerve nuclei, namely those of cranial nerves III, IV, V, VI, VII, VIII, IX, X, and XII in greater detail, as shown in Table 1 and Figures 3A–E. As such, the main associated functions are sensory, i.e., facial and taste, hearing, equilibrium, visceral thoracic and abdominal, chemoceptive and baroceptive, as well as motor, i.e., eye movements, pupil and lens function, chewing, facial expression, swallowing, speech, visceral thoracic and abdominal as well as tongue movements. The most intriguing and least explored role of the brainstem relates to its *integrative functions*. This is especially apparent from a behavioral point of view, given the brainstem’s involvement in complex state-dependent functions such as consciousness or emotion (Mesulam, 2000; Solms and Turnbull, 2002). Briefly, several integrative functions take place at the level of the midbrain, pons and medulla, dealing with regulation of consciousness and arousal (Moruzzi and Magoun, 1949; Solms and Turnbull, 2002; Edlow et al., 2012), autonomic visceral activity (Janig, 2008; Nieuwenhuys, 2008; Pattinson et al., 2009; Macefield and Henderson, 2010; Nolte et al., 2016), stress response (Feldman and Saphier, 1985; Feldman et al., 1995), pain (Eippert et al., 2009; Heinricher et al., 2009; Napadow et al., 2009; Linnman et al., 2012; Menant et al., 2016), and complex motor and sensory processing (Carpenter and Sutin, 1983; Guimaraes et al., 1998; Nolte et al., 2016). A critical role in integrative functions is served by the reticular formation, as reflected structurally by its diffuse pattern of connectivity with several other parts of the brain. Furthermore, cell groups that are sources of major neurotransmission systems in the brain are localized in the brainstem, namely the substantia nigra and ventral tegmental area (VTA; dopamine), locus coeruleus (noradrenaline), raphe nuclei (serotonin) and reticular formation (acetylcholine; e.g., Nieuwenhuys, 2012). All structures involved in the aforementioned functions were identified in the present study. It should be noted that the reticular formation and periaqueductal gray were labeled as single regions of interest, given the limitations in spatial resolution and contrast characteristics of our dataset for visualizing individual cell groups.

### Clinical Considerations

Alterations of brainstem structure have been and remain a current matter of relevance in neurological and neurosurgical clinical practice. Lesions of the brainstem such as embolic or hemorrhagic strokes and tumors can affect the conduit, cranial nerve, and integrative functions of the brainstem. In addition to classical neurological syndromes associated with cranial nerve alterations, such as Weber’s syndrome, medial medullary syndrome, and lateral medullary syndrome, which are diagnosed using the traditional neurological examination, other clinical conditions with brainstem involvement have been of recent interest. This is of particular note given the therapeutic interventions that can be applied using deep brain stimulation (DBS) for neuromodulation. Duret’s work on the involvement of the tegmental reticular formation, thalamic and extra-thalamic pathways as well as anterior forebrain circuits in alterations of consciousness has elucidated the role of lesions at the diencephalon-mesencephalic junction in coma and prolonged disorders of consciousness (Duret, 1955). Anatomical visualization of such structures and circuits is of clinical relevance given the therapeutic potential of neuromodulation of the brainstem (Fridman and Schiff, 2014). Interestingly, the cholinergic pedunculopontine tegmental nucleus, which projects to the thalamus and is involved in conscious behavior, is also involved in gait-balance control and may be a potential therapeutic target for postural and gait disorders in Parkinson’s disease (Wang et al., 2017). Neurochemical modulation of neurotransmitter systems arising in brainstem nuclei is of interest in neuropsychiatric practice given the involvement of transmitters in a variety of syndromes. More specifically, selective serotonin reuptake inhibitors (SSRIs) and selective norepinephrine reuptake inhibitors (SNRIs) are routinely used as antidepressants due to their ability to enhance the levels of these neurotransmitters, whereas dopamine antagonists have potent antipsychotic effects by blocking dopamine receptors (e.g., Schwartz et al., 2012). Furthermore, cholinergic agents are used in neurodegenerative conditions such as Alzheimer’s disease (e.g., Mesulam et al., 1989; Nolte et al., 2016).

### Limitations and Future Studies

Given the quality of the imaging dataset used herein, a cogent way of viewing limitations is by comparing these data with histological observations at the microscopic level. A critical student of neuroanatomy always has as the gold standard of analysis a microscopic level of explanation. Neuroimaging is still a few steps away, perhaps by a factor of 10 in spatial resolution, from reaching this level of visualization, although it seems this is rapidly approaching. In this study, we were able to delineate 25 nuclear masses and 16 fiber pathways in the human brainstem using a 50-micron spatial resolution in a postmortem human brainstem. This is still far from the detail provided by microscopic histological examination, where brainstem nuclei and fiber tracts have been identified and delineated using such techniques. It should be noted that in the present study the reticular formation was delineated as a region of interest by the exclusion of other structures and guided by histological atlases; the reticular formation *per se* could not be visualized with certainty using the current imaging methods. This limitation also applies to the description of the systems, such as the vestibuloocular system, which has more connections than were visualized in the current dataset. We recognize as well that certain boundaries of the ROIs segmented in this investigation may change as the state of MRI technology and neuroanatomical knowledge grows. We will publish updates in an on-line repository as the present structural borders may be modified as we receive feedback.

Future studies using combined high-resolution MRI datasets with their histological sections for comparison (Makris et al., 2013) should provide further validation and accuracy of anatomical delineations in brainstem anatomy. These approaches will also permit a formal evaluation of the variability in volume and extent of regions of the human brainstem. To date, such variability in the human brainstem has been incompletely studied (Andrew and Watkins, 1969; Afshar, 1978; Blood et al., 2012); more detailed knowledge will be a critical step in understanding normal and abnormal brainstem anatomy.

As MRI technology advances it is expected that higher resolution datasets than the one used in the present study will become available. Thus, histological analyses should be done in these samples as well in a co-registered fashion with the MRI datasets for precise and valid comparisons. In addition to the greater level of structural detail that these types of comparison will produce, they are also expected to generate a more complete description of brainstem systems.

## Conclusions

Using a high-resolution MRI postmortem dataset of the human brainstem and the 3D Slicer platform set of tools for image analysis, we were able to identify and delineate a large number of nuclear masses, the largest so far to our knowledge. Also, we identified and delineated most of the sizeable brainstem white matter fiber tracts of conduit and interconnecting nature.

## Data Availability Statement

All datasets presented in this study are included in the article/Supplementary Material.

## Author Contributions

NM, RK, EY, RR, EC, and GJ conceived the project. NM, RR, PW-B, IN, and GP analyzed the data and prepared the figures. NM wrote the first draft of the manuscript, which was read and revised by all the authors.

## Conflict of Interest

The authors declare that the research was conducted in the absence of any commercial or financial relationships that could be construed as a potential conflict of interest.

## Footnotes

^1^Note that the neuroanatomical definition of pons refers strictly to the ventral protrusion of the metencephalon and does not include the pontine tegmental nuclei (the pons Varoli, see ten Donkelaar et al., 2018).
