# Supplementary material for: 3D Exploration of the Brainstem in 50-Micron Resolution MRI
Source: Front Neuroanat. 2020 Sep 23;14:40. doi: 10.3389/fnana.2020.00040 (PMC7538715; doi:10.3389/fnana.2020.00040)
Supplement: Supplementary file 1 [file Data_Sheet_2.PDF]

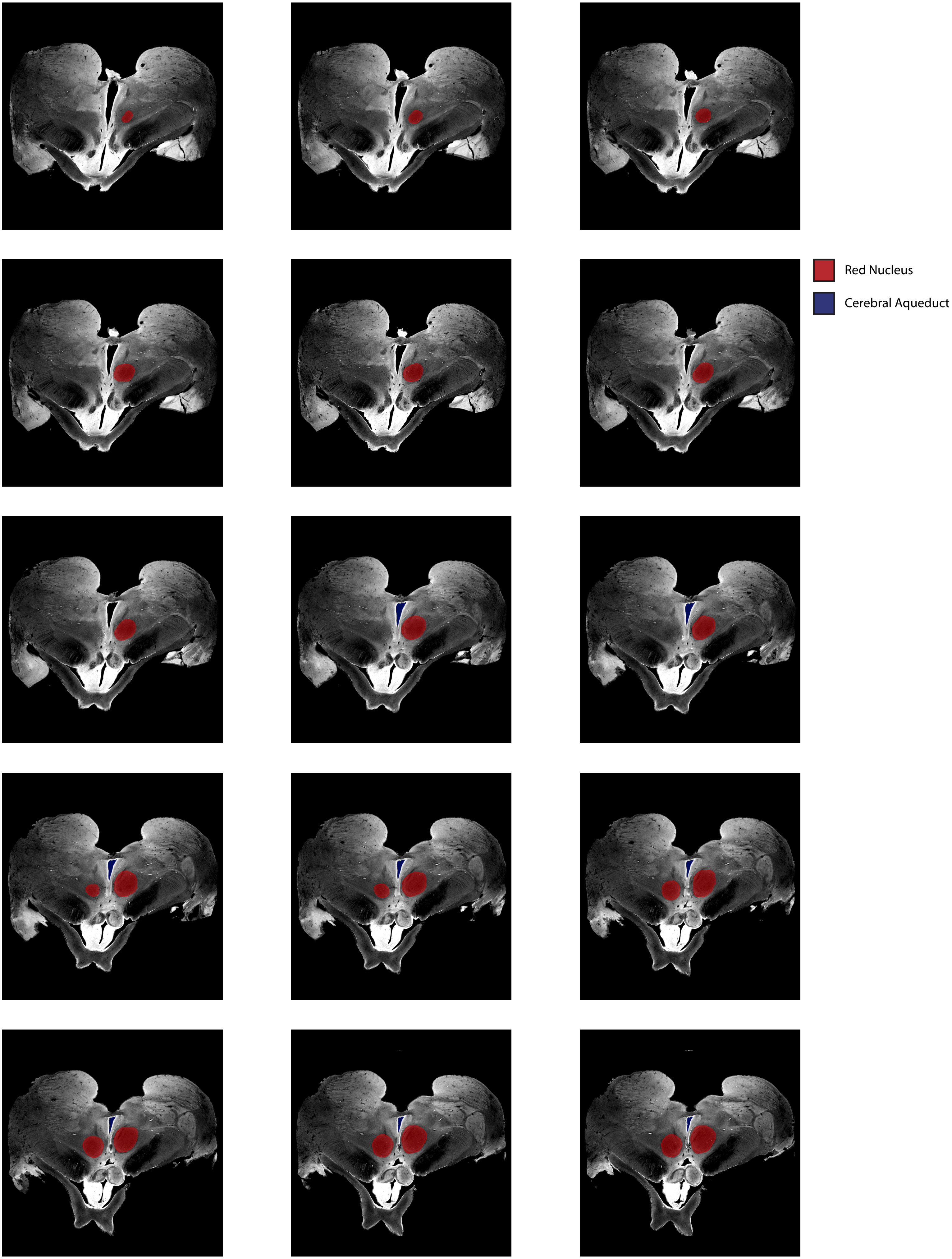
Red Nucleus
Cerebral Aqueduct

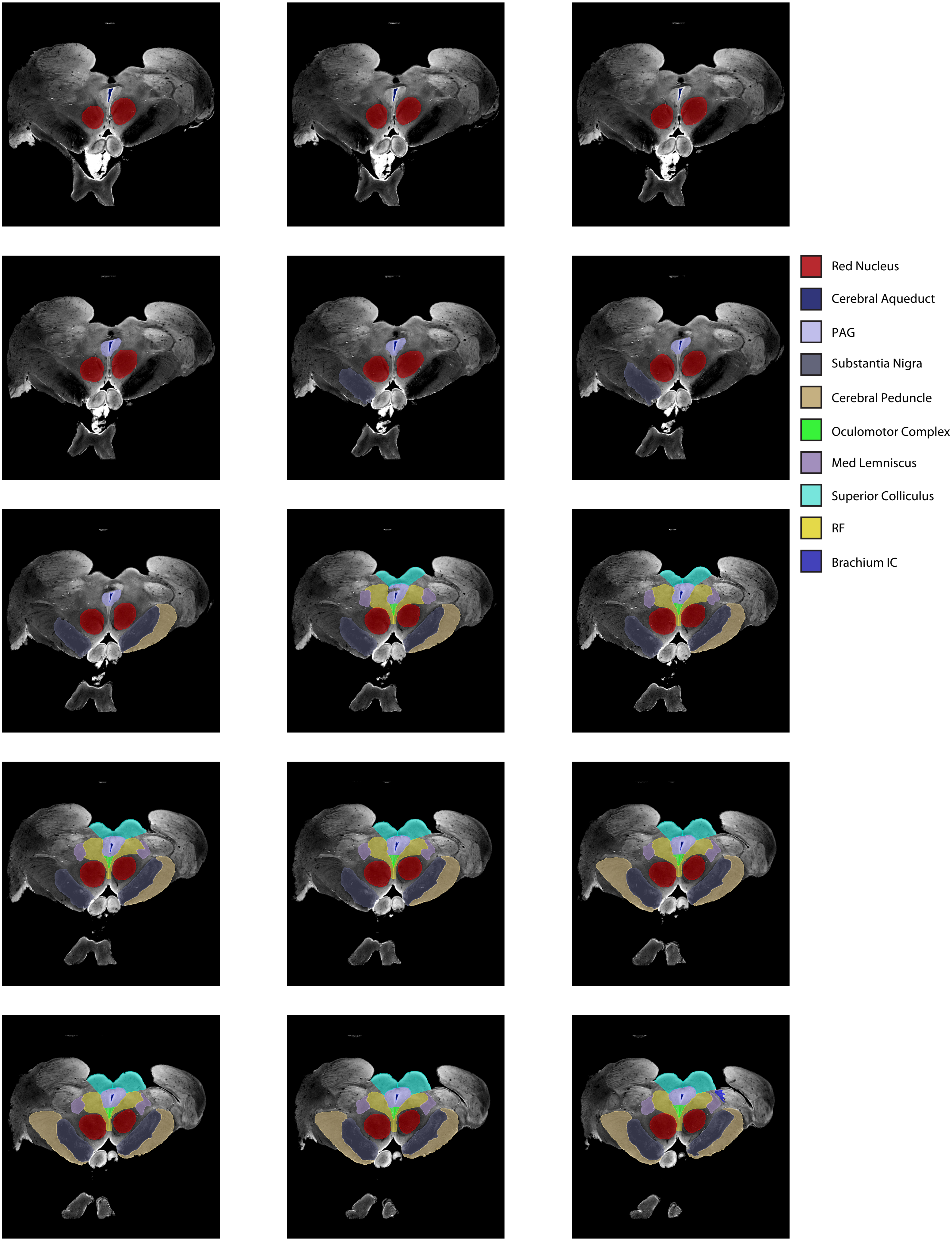

Red Nucleus
Cerebral Aqueduct
PAG
Substantia Nigra
Cerebral Peduncle
Oculomotor Complex
Med Lemniscus
Superior Colliculus
RF
Brachium IC

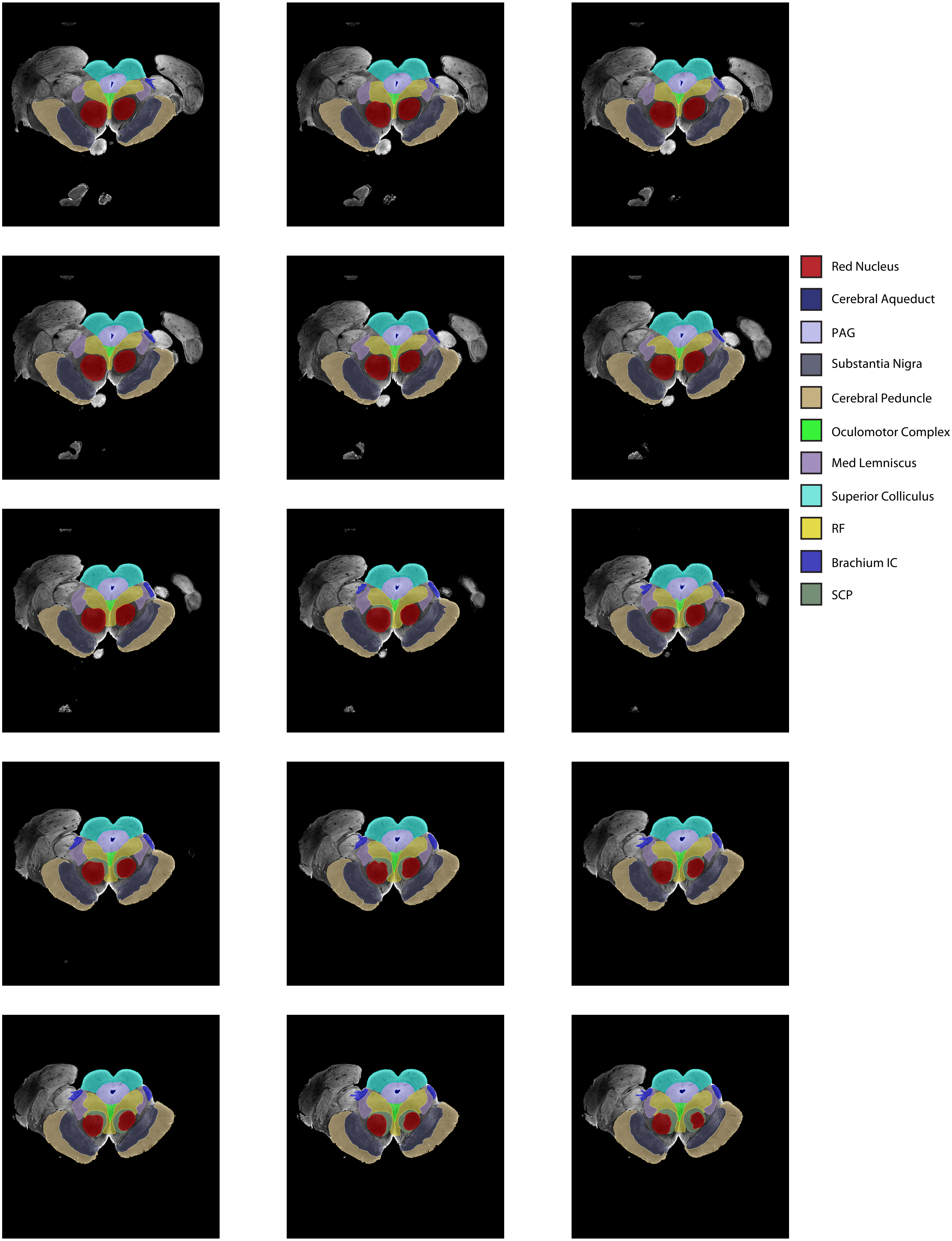
Red Nucleus
Cerebral Aqueduct
PAG
Substantia Nigra
Cerebral Peduncle
Oculomotor Complex
Med Lemniscus
Superior Colliculus
RF
Brachium IC
SCP
