# Supplementary material for: 3D Exploration of the Brainstem in 50-Micron Resolution MRI
Source: Front Neuroanat. 2020 Sep 23;14:40. doi: 10.3389/fnana.2020.00040 (PMC7538715; doi:10.3389/fnana.2020.00040)
Supplement: Supplementary file 2 [file Data_Sheet_3.PDF]

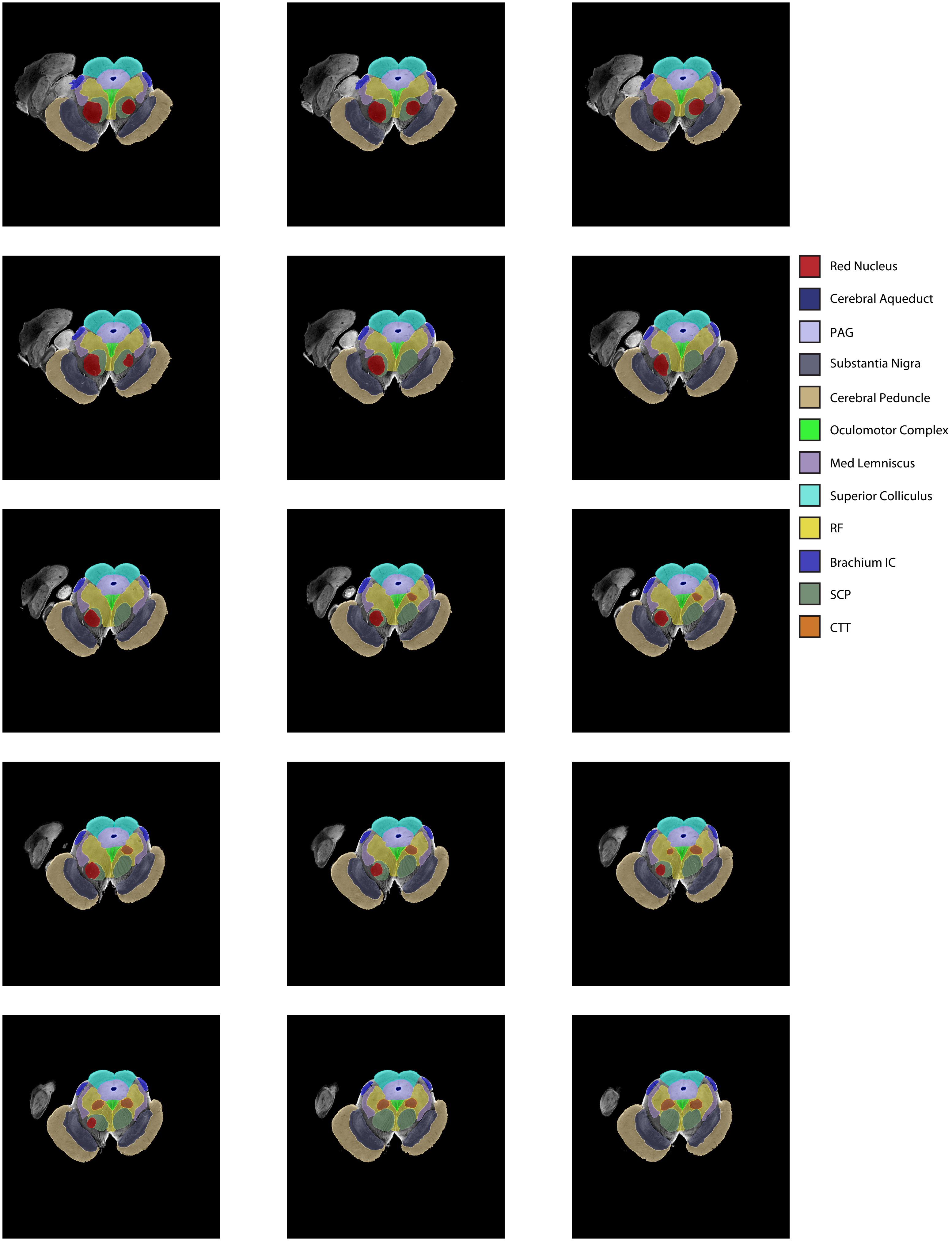
Red Nucleus
Cerebral Aqueduct
PAG
Substantia Nigra
Cerebral Peduncle
Oculomotor Complex
Med Lemniscus
Superior Colliculus
RF
Brachium IC
SCP
CTT

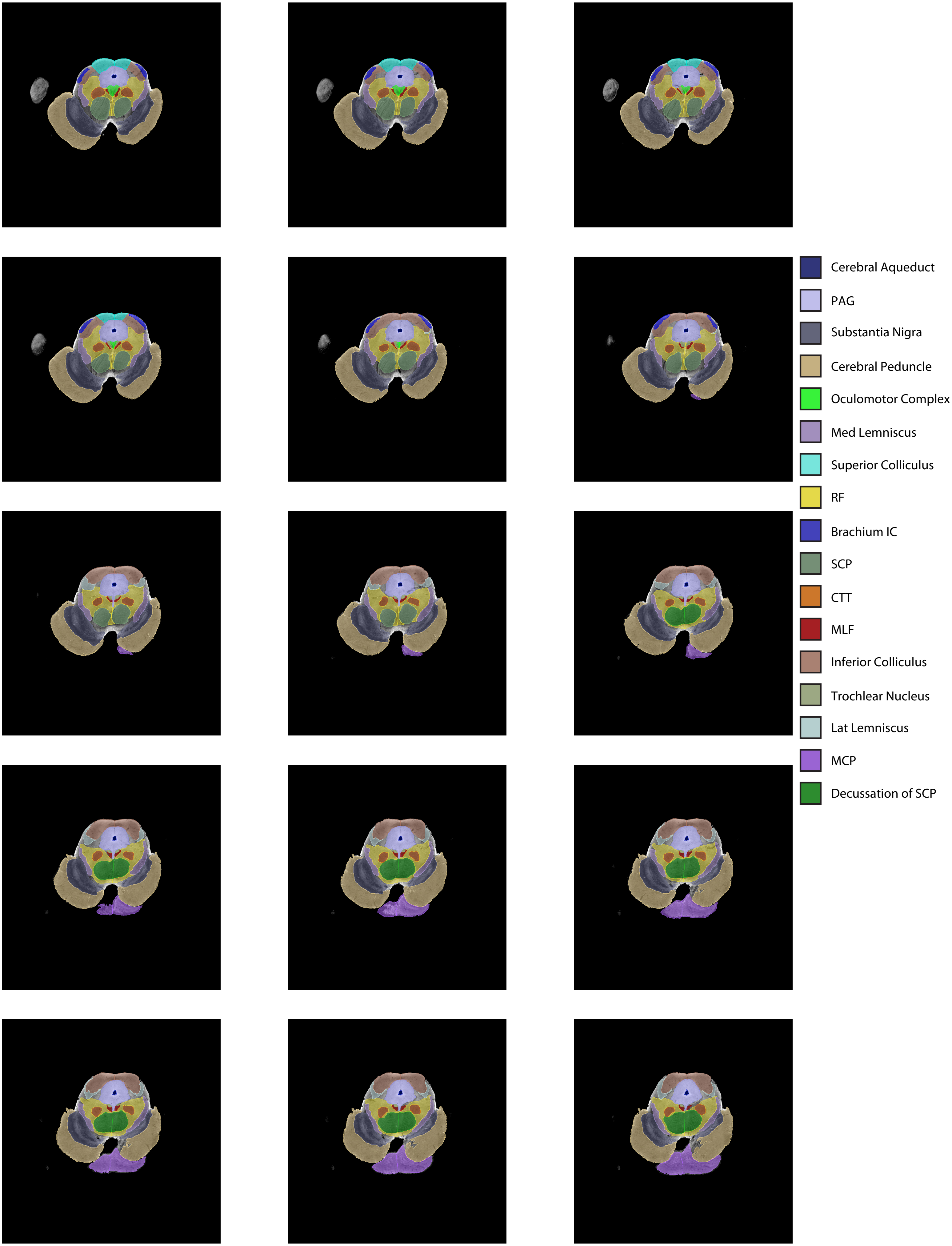
Cerebral Aqueduct
PAG
Substantia Nigra
Cerebral Peduncle
Oculomotor Complex
Med Lemniscus
Superior Colliculus
RF
Brachium IC
SCP
CTT
MLF
Inferior Colliculus
Trochlear Nucleus
Lat Lemniscus
MCP
Decussation of SCP

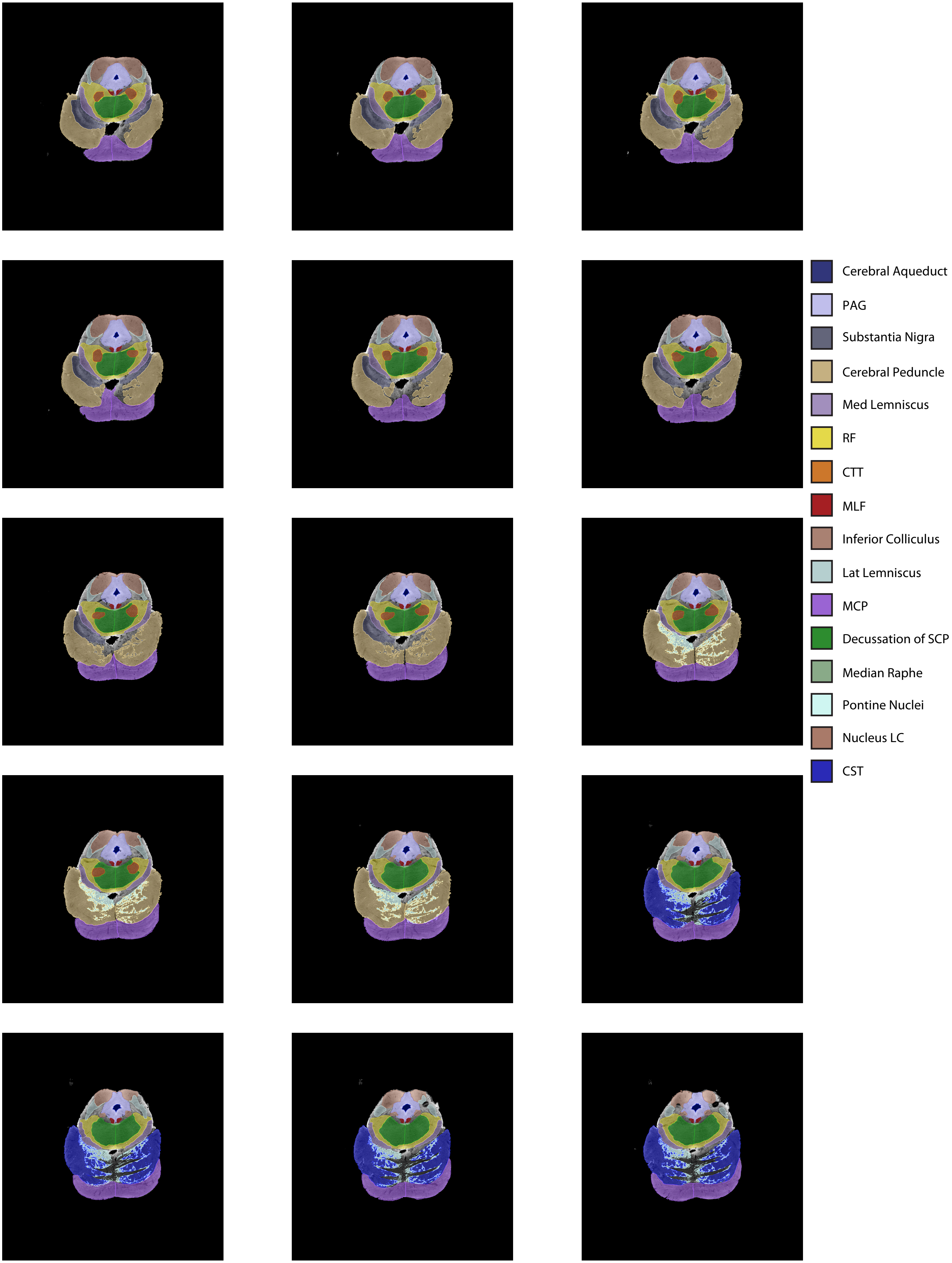
Cerebral Aqueduct
PAG
Substantia Nigra
Cerebral Peduncle
Med Lemniscus
RF
CTT
MLF
Inferior Colliculus
Lat Lemniscus
MCP
Decussation of SCP
Median Raphe
Pontine Nuclei
Nucleus LC
CST

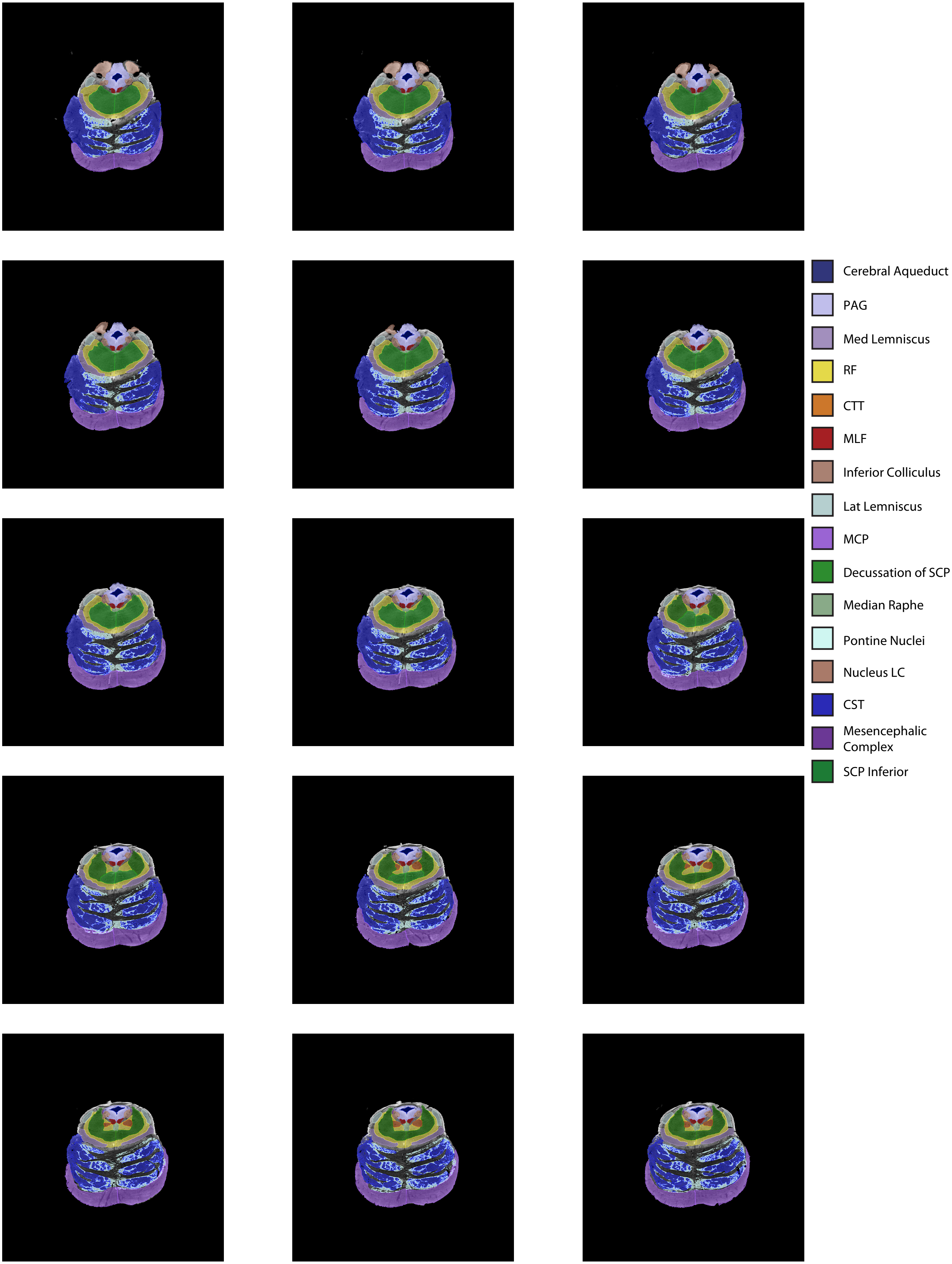
Cerebral Aqueduct
PAG
Med Lemniscus
RF
CTT
MLF
Inferior Colliculus
Lat Lemniscus
MCP
Decussation of SCP
Median Raphe
Pontine Nuclei
Nucleus LC
CST
Mesencephalic Complex
SCP Inferior

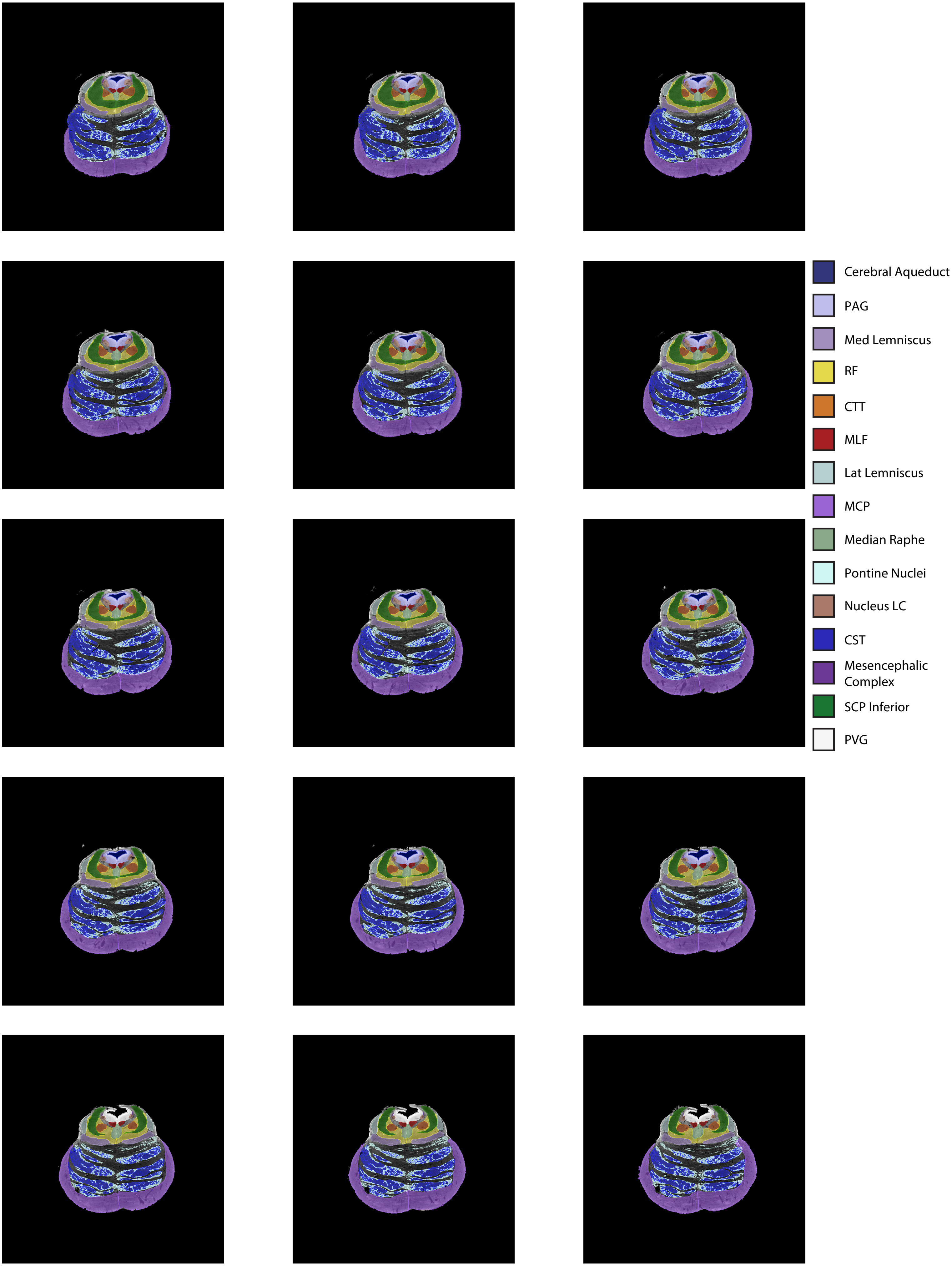
Cerebral Aqueduct
PAG
Med Lemniscus
RF
CTT
MLF
Lat Lemniscus
MCP
Median Raphe
Pontine Nuclei
Nucleus LC
CST
Mesencephalic Complex
SCP Inferior
PVG
