# Supplementary material for: 3D Exploration of the Brainstem in 50-Micron Resolution MRI
Source: Front Neuroanat. 2020 Sep 23;14:40. doi: 10.3389/fnana.2020.00040 (PMC7538715; doi:10.3389/fnana.2020.00040)
Supplement: Supplementary file 3 [file Data_Sheet_4.PDF]

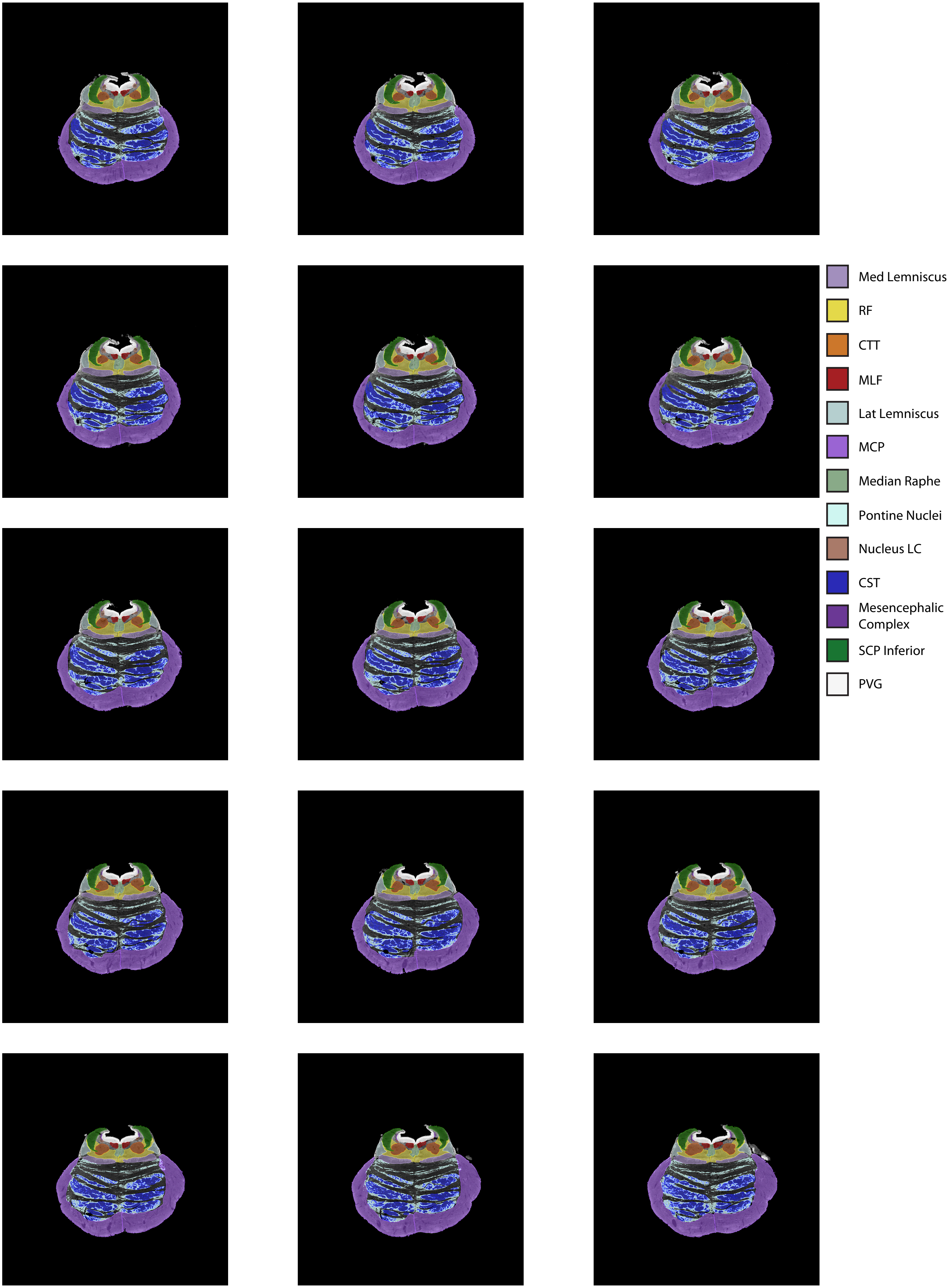
Med Lemniscus
RF
CTT
MLF
Lat Lemniscus
MCP
Median Raphe
Pontine Nuclei
Nucleus LC
CST
Mesencephalic Complex
SCP Inferior
PVG

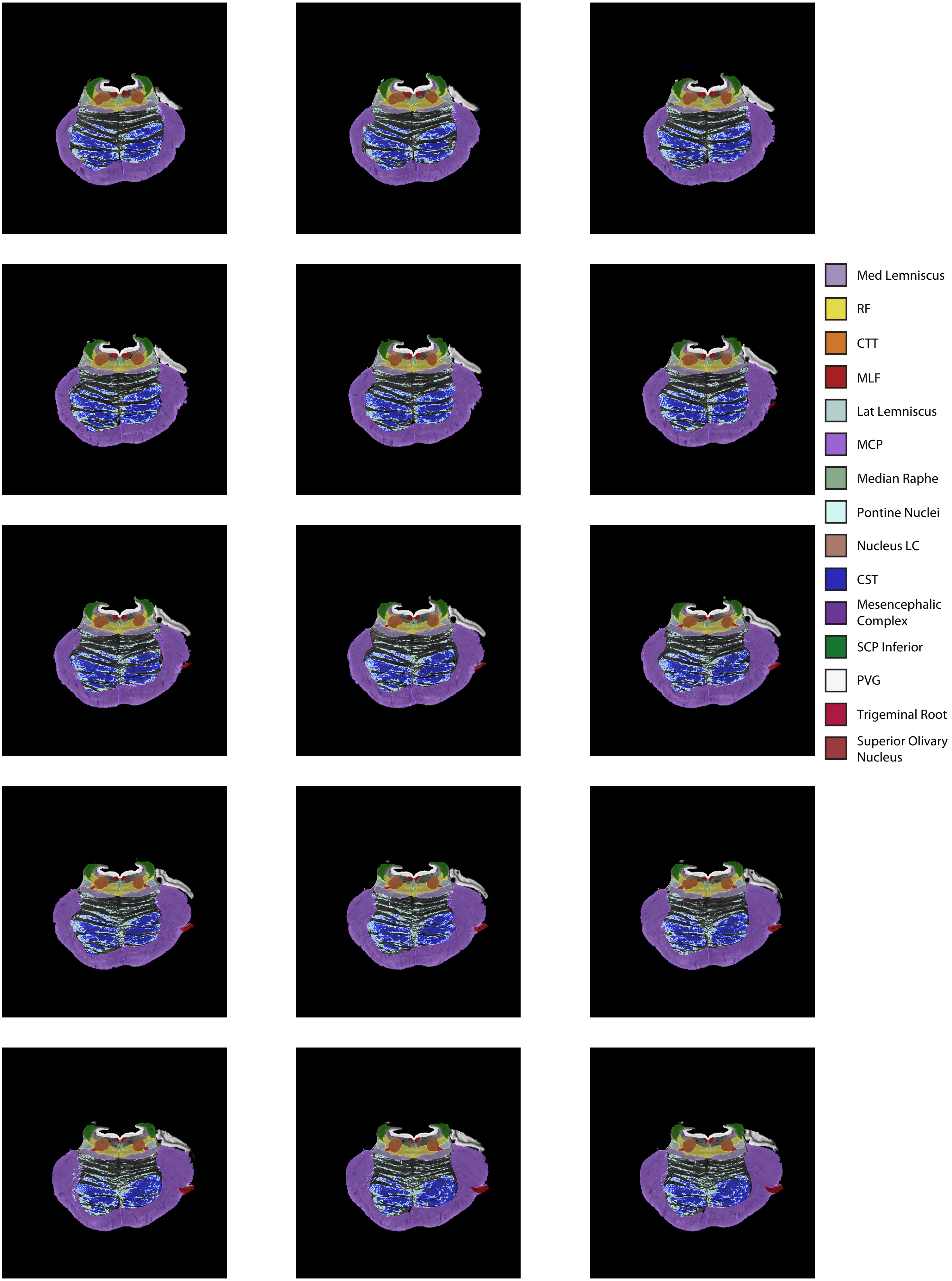

Med Lemniscus
RF
CTT
MLF
Lat Lemniscus
MCP
Median Raphe
Pontine Nuclei
Nucleus LC
CST
Mesencephalic Complex
SCP Inferior
PVG
Trigeminal Root
Superior Olivary Nucleus

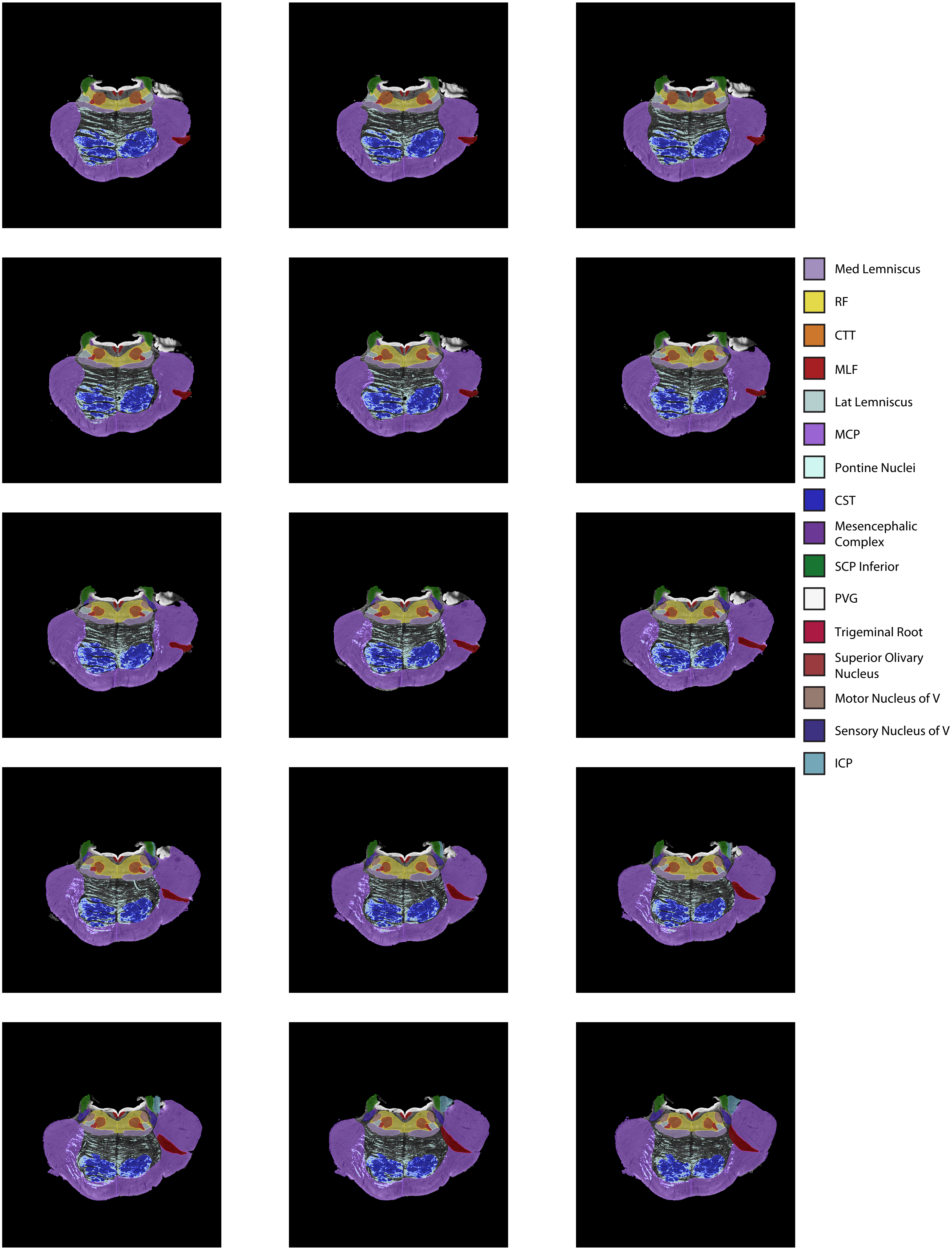
Med Lemniscus
RF
CTT
MLF
Lat Lemniscus
MCP
Pontine Nuclei
CST
Mesencephalic Complex
SCP Inferior
PVG
Trigeminal Root
Superior Olivary Nucleus
Motor Nucleus of V
Sensory Nucleus of V
ICP

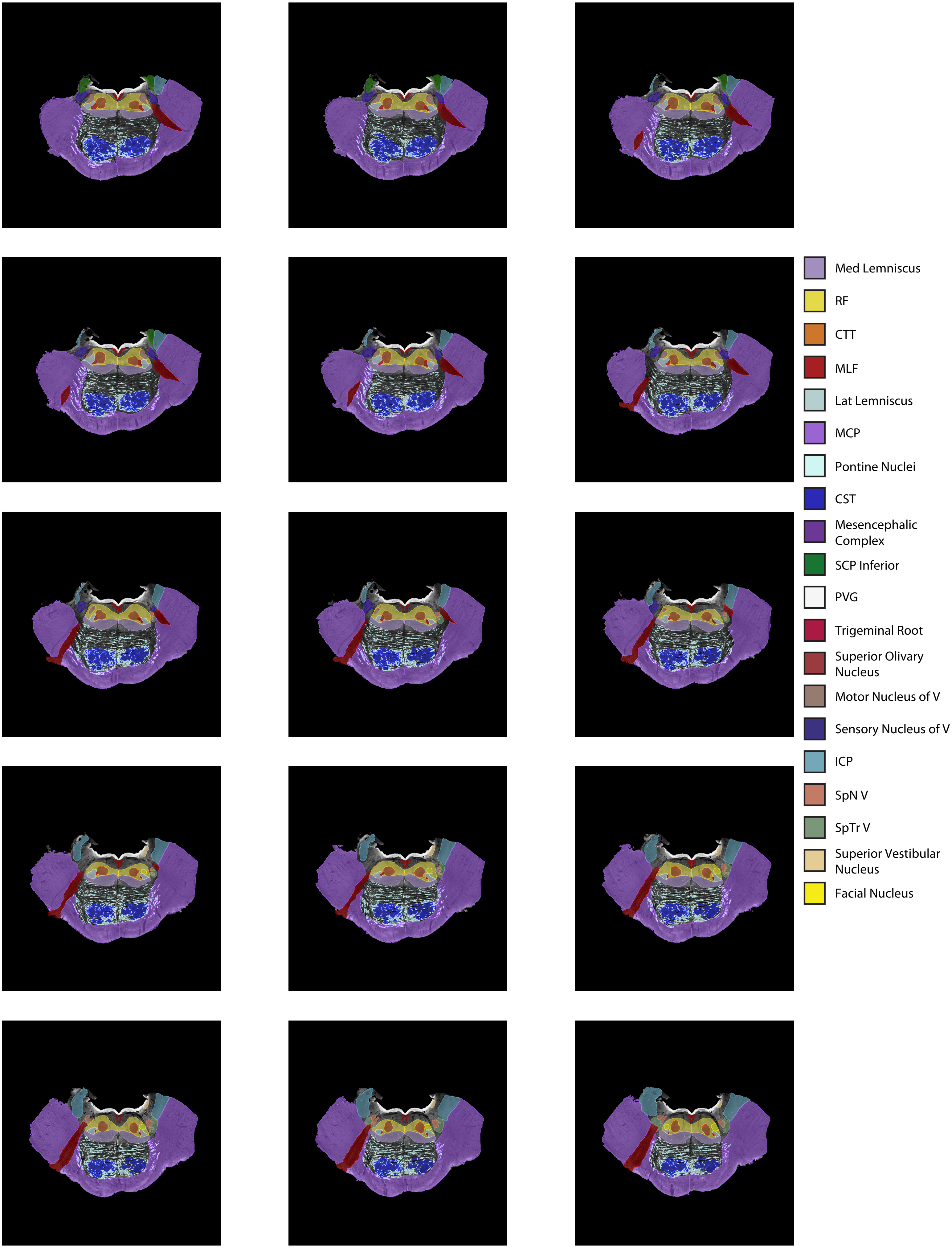
Med Lemniscus
RF
CTT
MLF
Lat Lemniscus
MCP
Pontine Nuclei
CST
Mesencephalic Complex
SCP Inferior
PVG
Trigeminal Root
Superior Olivary Nucleus
Motor Nucleus of V
Sensory Nucleus of V
ICP
SpN V
SpTr V
Superior Vestibular Nucleus
Facial Nucleus
