# Supplementary material for: 3D Exploration of the Brainstem in 50-Micron Resolution MRI
Source: Front Neuroanat. 2020 Sep 23;14:40. doi: 10.3389/fnana.2020.00040 (PMC7538715; doi:10.3389/fnana.2020.00040)
Supplement: Supplementary file 4 [file Data_Sheet_5.PDF]

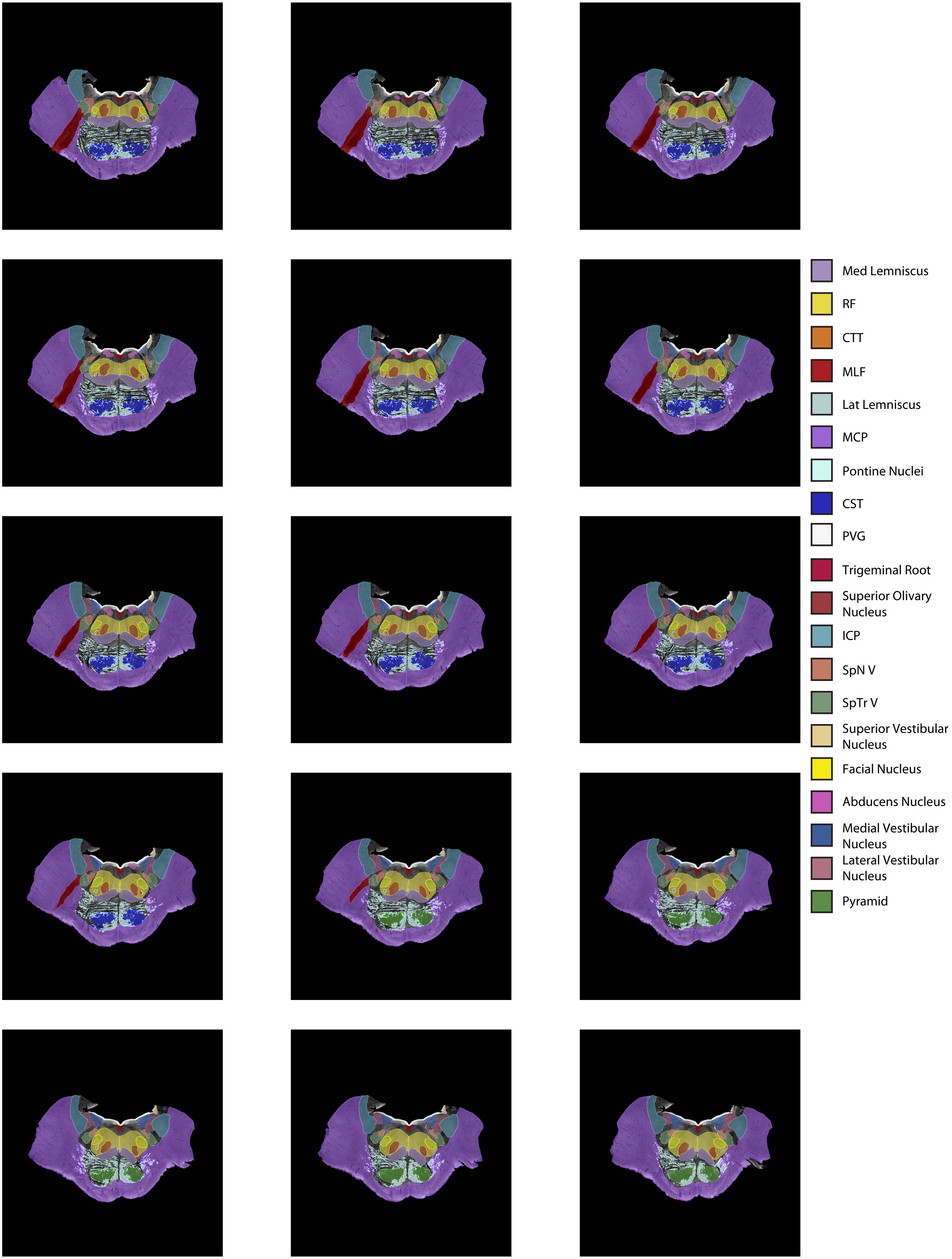
Med Lemniscus
RF
CTT
MLF
Lat Lemniscus
MCP
Pontine Nuclei
CST
PVG
Trigeminal Root
Superior Olivary Nucleus
ICP
SpN V
SpTr V
Superior Vestibular Nucleus
Facial Nucleus
Abducens Nucleus
Medial Vestibular Nucleus
Lateral Vestibular Nucleus
Pyramid

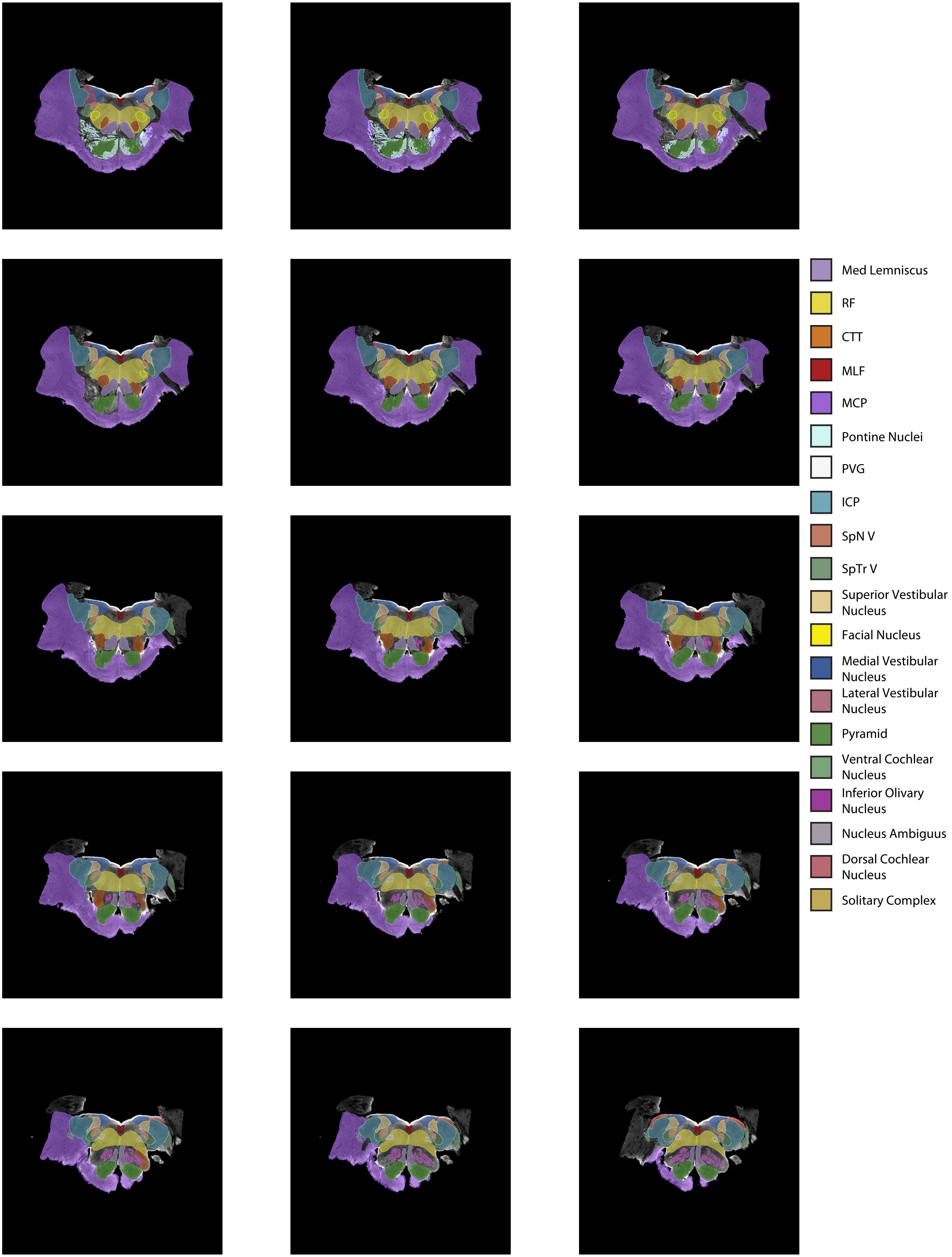

Med Lemniscus
RF
CTT
MLF
MCP
Pontine Nuclei
PVG
ICP
SpN V
SpTr V
Superior Vestibular Nucleus
Facial Nucleus
Medial Vestibular Nucleus
Lateral Vestibular Nucleus
Pyramid
Ventral Cochlear Nucleus
Inferior Olivary Nucleus
Nucleus Ambiguus
Dorsal Cochlear Nucleus
Solitary Complex

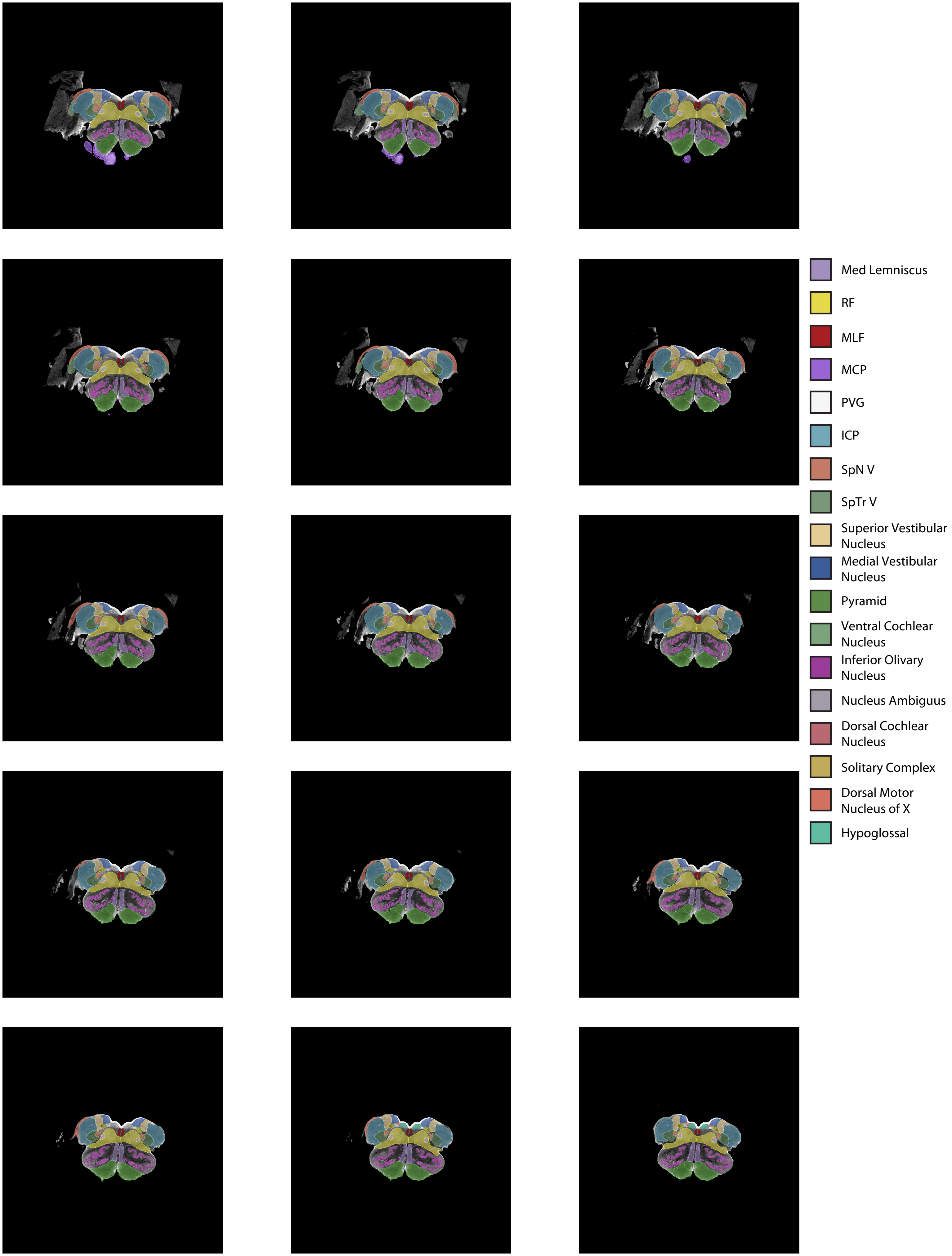
Med Lemniscus
RF
MLF
MCP
PVG
ICP
SpN V
SpTr V
Superior Vestibular Nucleus
Medial Vestibular Nucleus
Pyramid
Ventral Cochlear Nucleus
Inferior Olivary Nucleus
Nucleus Ambiguus
Dorsal Cochlear Nucleus
Solitary Complex
Dorsal Motor Nucleus of X
Hypoglossal

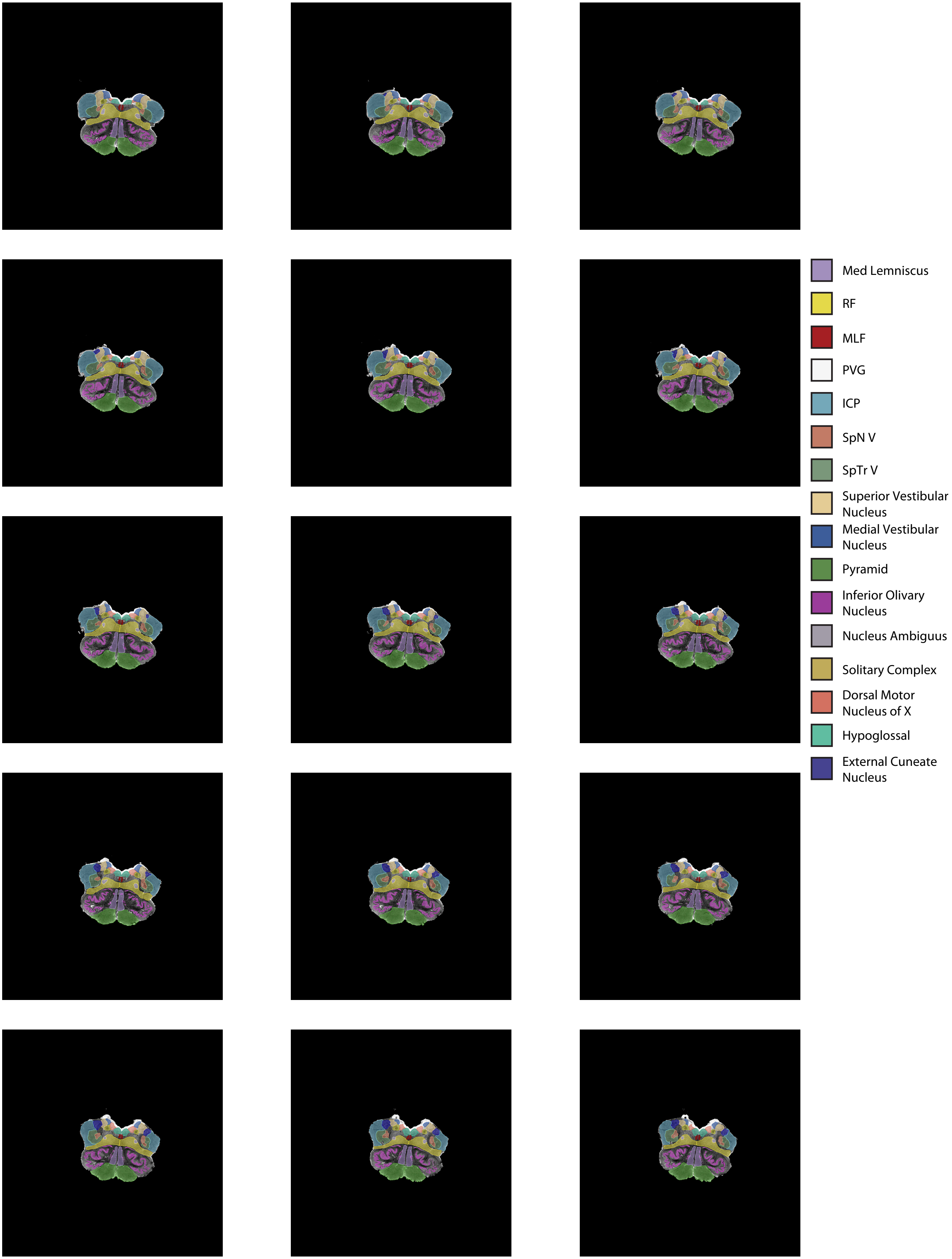

Med Lemniscus
RF
MLF
PVG
ICP
SpN V
SpTr V
Superior Vestibular Nucleus
Medial Vestibular Nucleus
Pyramid
Inferior Olivary Nucleus
Nucleus Ambiguus
Solitary Complex
Dorsal Motor Nucleus of X
Hypoglossal
External Cuneate Nucleus

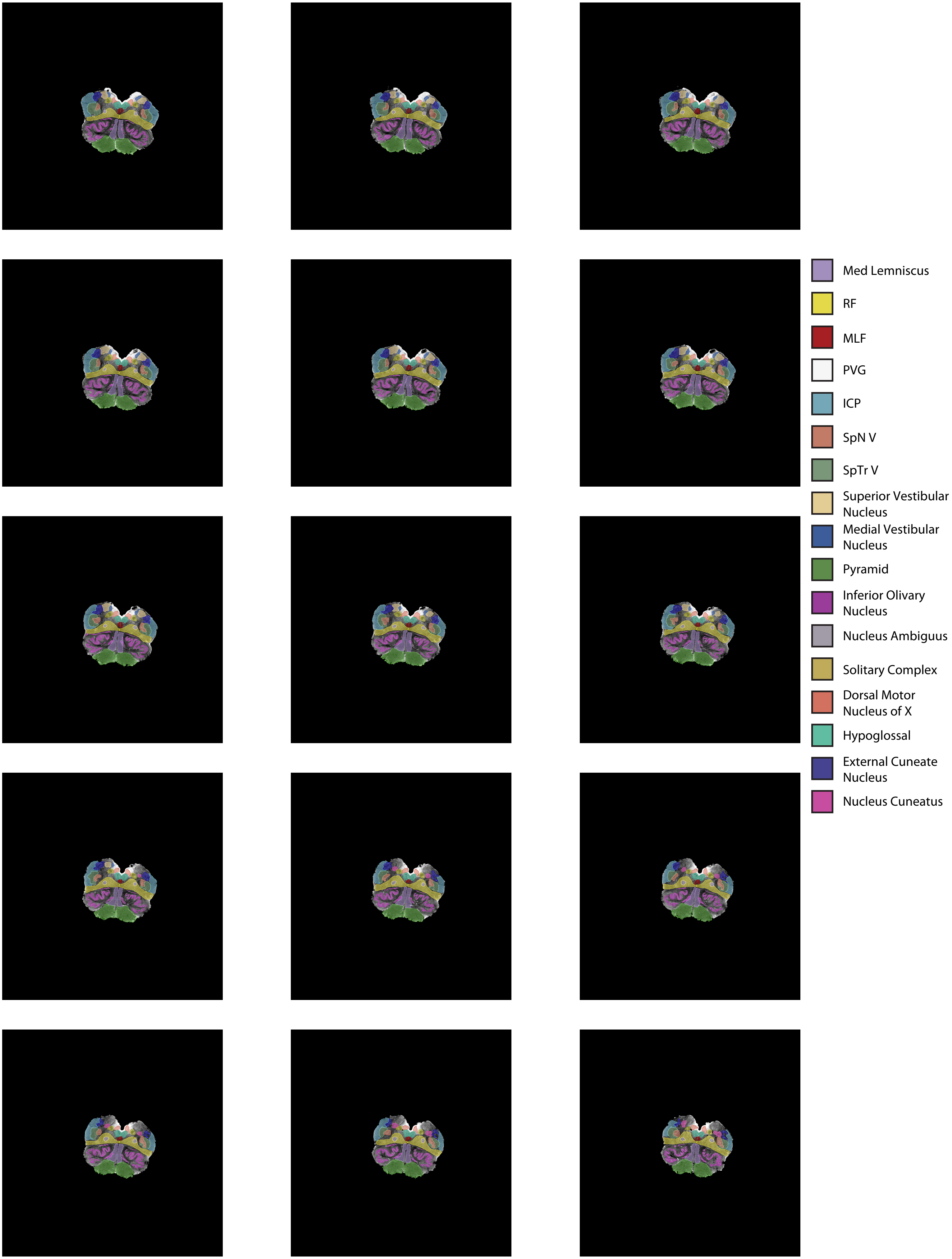
Med Lemniscus
RF
MLF
PVG
ICP
SpN V
SpTr V
Superior Vestibular Nucleus
Medial Vestibular Nucleus
Pyramid
Inferior Olivary Nucleus
Nucleus Ambiguus
Solitary Complex
Dorsal Motor Nucleus of X
Hypoglossal
External Cuneate Nucleus
Nucleus Cuneatus

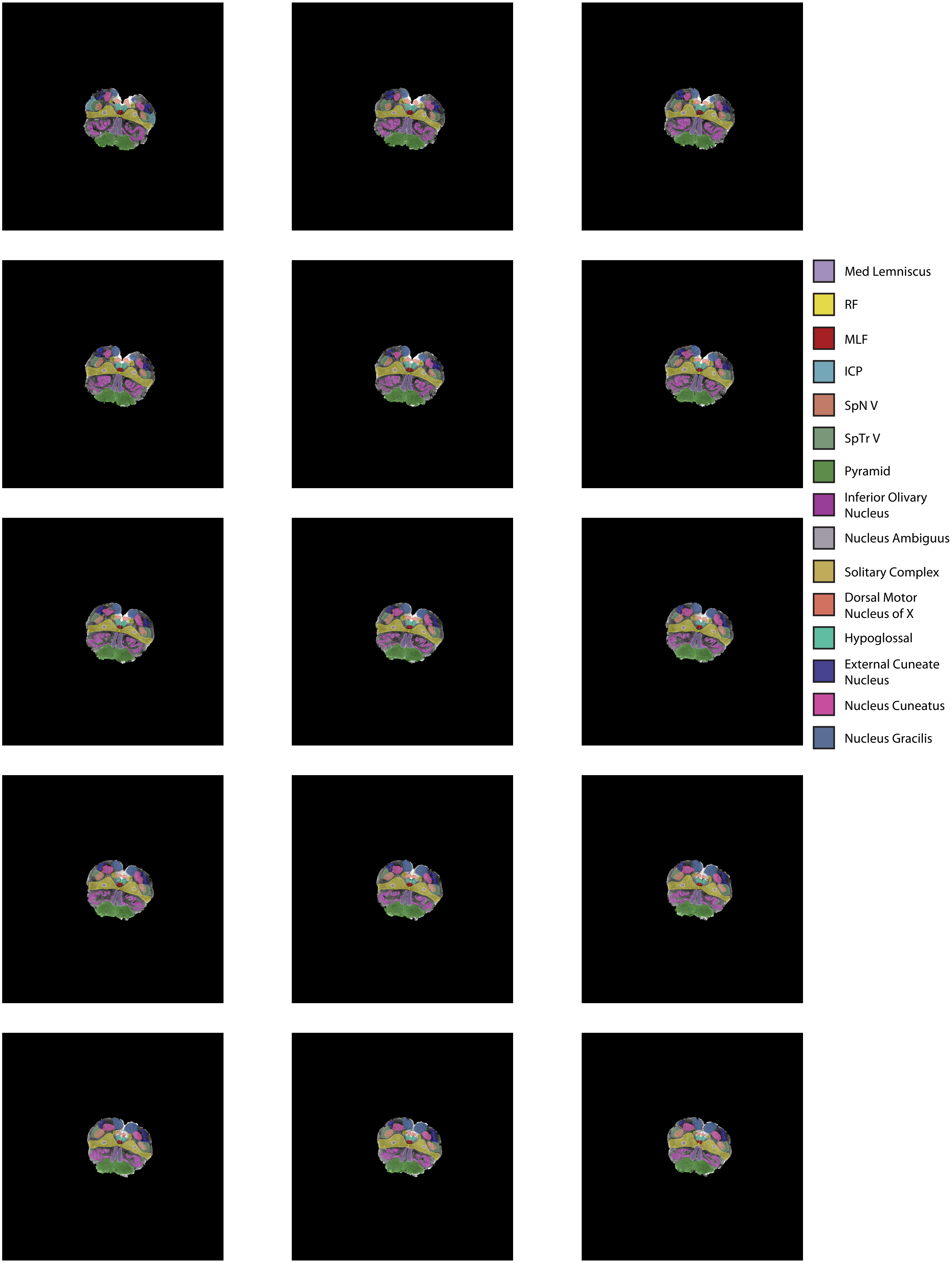
Med Lemniscus
RF
MLF
ICP
SpN V
SpTr V
Pyramid
Inferior Olivary Nucleus
Nucleus Ambiguus
Solitary Complex
Dorsal Motor Nucleus of X
Hypoglossal
External Cuneate Nucleus
Nucleus Cuneatus
Nucleus Gracilis

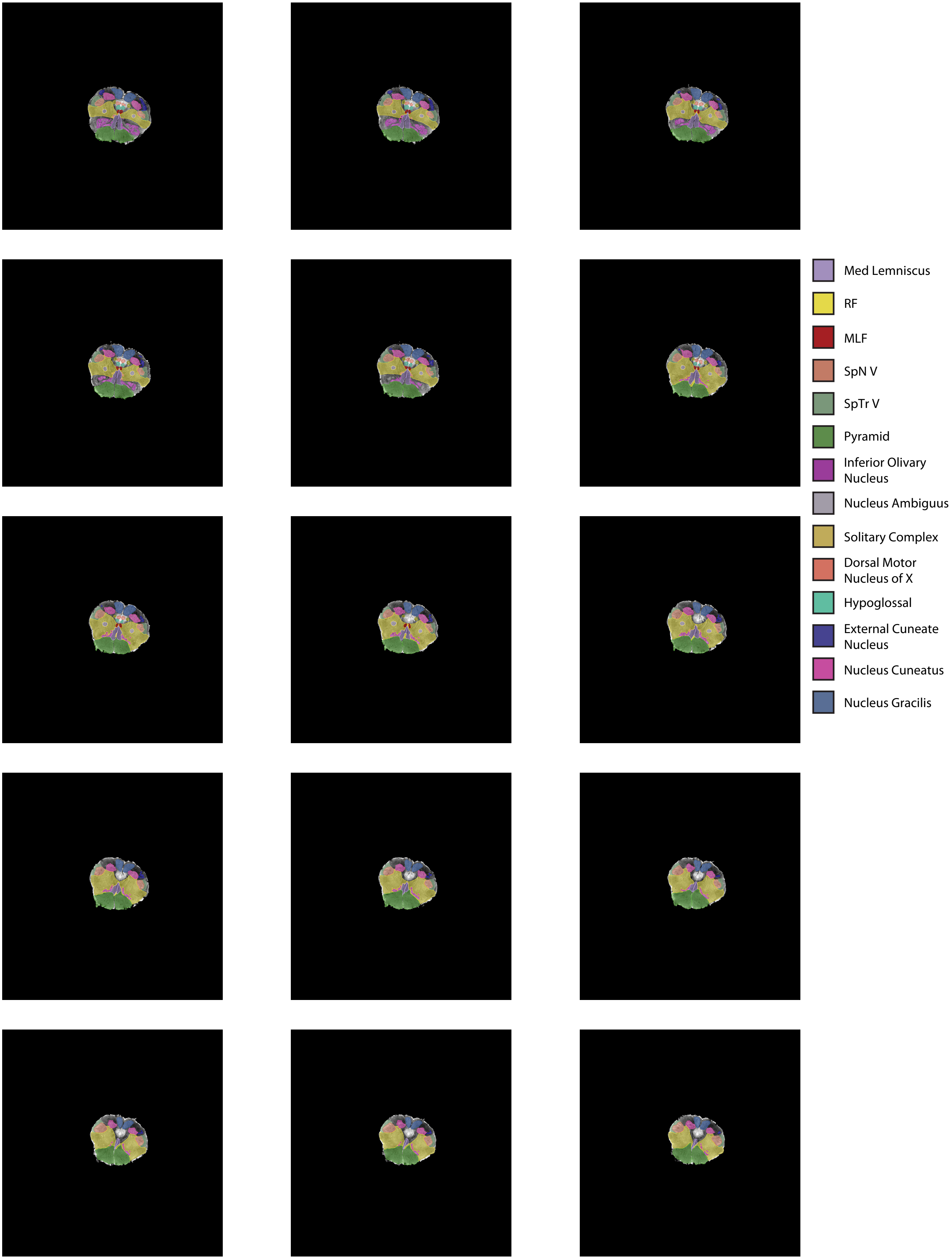
Med Lemniscus
RF
MLF
SpN V
SpTr V
Pyramid
Inferior Olivary Nucleus
Nucleus Ambiguus
Solitary Complex
Dorsal Motor Nucleus of X
Hypoglossal
External Cuneate Nucleus
Nucleus Cuneatus
Nucleus Gracilis

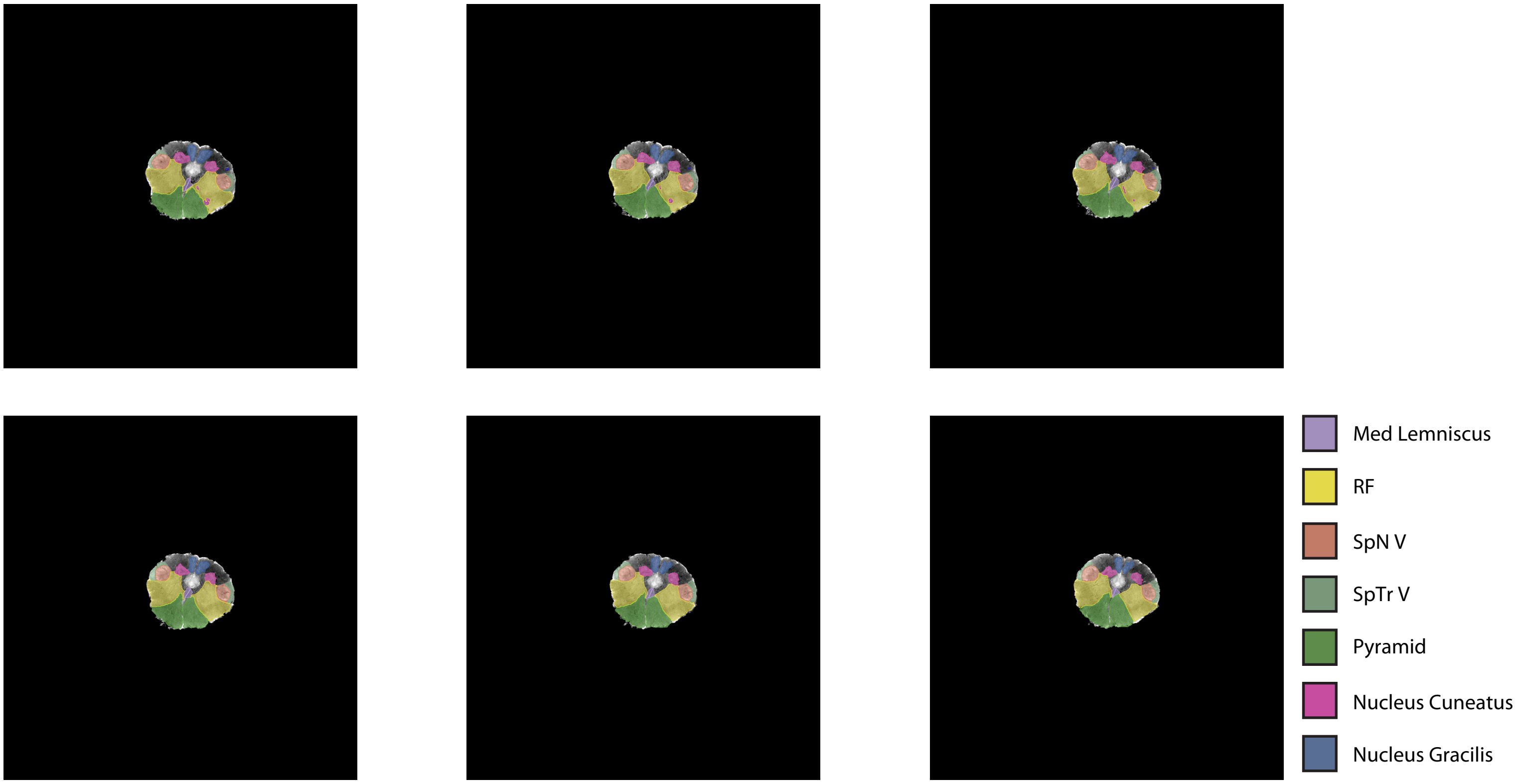
Med Lemniscus
RF
SpN V
SpTr V
Pyramid
Nucleus Cuneatus
Nucleus Gracilis
